# Belief inducibility and informativeness

**DOI:** 10.1007/s11238-023-09963-7

**Published:** 2023-12-24

**Authors:** P. Jean-Jacques Herings, Dominik Karos, Toygar T. Kerman

**Affiliations:** 1 Department of Econometrics and Operations Research, Tilburg University, P.O. Box 90153, 5000 LE Tilburg, The Netherlands; 2 Center for Mathematical Economics, Bielefeld University, P.O. Box 100131, 33501 Bielefeld, Germany; 3 Institute of Economics, Corvinus University of Budapest, Fővam tér 8, Budapest, 1093 Hungary

**Keywords:** Game theory, Information design, Inducible distributions, Informativeness

## Abstract

We consider a group of receivers who share a common prior on a finite state space and who observe private correlated messages that are contingent on the true state of the world. Our focus lies on the beliefs of receivers induced via the signal chosen by the sender and we provide a comprehensive analysis of the inducible distributions of posterior beliefs. Classifying signals as minimal, individually minimal, and language-independent, we show that any inducible distribution can be induced by a language-independent signal. We investigate the role of the different classes of signals for the amount of higher order information that is revealed to receivers. The least informative signals that induce a fixed distribution over posterior belief profiles lie in the relative interior of the set of all language-independent signals inducing that distribution.

## Introduction

In any economic model which involves a group of agents whose decisions depend on their posterior beliefs, one of the essential questions is “what distributions over posterior beliefs of agents can be induced?" In their seminal paper, Kamenica and Gentzkow (2011) consider communication between a sender and a receiver who share a common prior and show that the only restriction on the set of inducible distributions over posterior belief profiles is Bayes plausibility: the expected posterior belief is equal to the prior.^1^ It follows from their insight that Bayes plausibility and identical beliefs are necessary and sufficient in the case of multiple receivers and *public* communication, that is, when messages are perfectly correlated. Yet, in this case the set of inducible distributions over posteriors is very limited since all receivers have the same ex post belief. In the present paper we are interested in private communication, which, in contrast, enables the sender to achieve a richer belief space. It is straightforward to verify that Bayes plausibility is not sufficient to ensure inducibility in such setups; this raises the first question we tackle in the paper: providing a characterization of the set of inducible posterior beliefs under private communication.

Another aspect which is important for both the sender and receivers is the *informativeness* of a signal. In the original information design setup introduced by Aumann et al. (1995), the authors were interested in communication that reveals as little private information as possible. In our paper, a signal realization does not only reveal information about the true state of the world: as there are multiple receivers who each obtain a private message, it also induces information partitions that determine what any receiver knows about another receiver’s knowledge of the true state and the signal realization. Thus, we compare the informativeness of signals in terms of “knowledge" in the sense of Hintikka (1962). To be more precise, we compare information sets induced by a signal, which are similar to elements of information partitions in Aumann (1976). The second main question we answer is: what types of signals are the least informative? We provide a characterization for least informative signals that induce a given posterior distribution.

We consider a sender who commits to a signal that sends private correlated *messages* to the receivers. Receivers know the joint distribution of *message profiles*, but they only observe their own private message from the message profile realization. We first show that there are posterior belief profiles, which the sender cannot achieve with positive probability. More precisely, for a given posterior belief profile, there exists a signal that induces a distribution which puts positive weight on it if and only if there exists a state which is deemed possible by all receivers according to this belief profile. As an example, consider an operative who follows Machiavelli’s advice *divide et impera* and, thus, wants to create political unrest in a foreign country by implementing a very heterogeneous belief profile.^2^ Suppose that there are only two states, say *X* and *Y*. Then it is impossible for the operative to implement a distribution that puts positive weight on a posterior belief profile in which one receiver believes the state is *X* with probability 1 and another receiver believes that the state is *Y* with probability 1. At the same time, a posterior belief profile in which the first receiver’s belief that the state is *X* is equal to 1, and the second receiver’s belief is arbitrarily close to 0 can be achieved with positive probability.

We next define particular classes of signals. We first consider *minimal* signals under which distinct message profiles lead to distinct posterior belief profiles. While this ensures that no two message profiles implement the same posterior belief profile, there might still be individual receivers for whom different messages lead to the same posterior. If for each receiver every posterior is induced by a unique message, the signal is called *individually minimal*. If, additionally, the sent messages are themselves posteriors such that each message induces itself, we call the signals *language-independent* (LIS). Here, a sender simply tells the receivers what belief they should have, and the messages are sent with probabilities such that receivers will believe the message. We show that restricting attention to language-independent signals is without loss of generality, that is, if a posterior distribution can be induced, it can be induced by an LIS.

As mentioned before, in the presence of multiple receivers Bayes plausibility is necessary but not sufficient for a distribution to be inducible. We characterize the set of inducible distributions of posteriors by showing that a Bayes-plausible distribution is inducible if and only if there exists a non-negative matrix *p* with dimensions equal to the number of states and the number of posterior belief profiles, respectively, which satisfies a particular system of linear equations. In particular, the set of matrices that satisfy these equations is a convex polytope, which implies that the set of language-independent signals that induce a given distribution over posterior belief profiles is a convex polytope as well.

Once we determine whether a distribution of posterior beliefs is inducible, we explore the informativeness of different signals which induce it. Since messages can be correlated, the message a receiver obtains reveals not only information about the true state of the world, but also about the information that other receivers have. Let us return to our operative who wants to create chaos in a foreign country. If one receiver knew that another receiver knew whether the true state is *X* or *Y*, he might decide not to engage in an argument at all. Thus, our operative might want to reveal as little information as possible to any receiver about what other receivers know. As an example suppose that before the operative engages, two receivers believe that either state might be true with probability 1/2. Suppose the operative engages in private communication with both and sends message profiles as follows.

**Table:** 

$\pi$	(*v*, *x*)	(*v*, *y*)	(*w*, *w*)
*X*	$\frac{1}{2}$	0	$\frac{1}{2}$
*Y*	0	$\frac{1}{2}$	$\frac{1}{2}$

The first and second rows of the table reflect the probabilities by which the message profiles are sent conditional on the state being *X* and *Y*, respectively. In this case, receiver 2 knows that the true state is *X* if he observes *x*, he knows the true state is *Y* if he observes *y*, and he learns nothing if he observes *w*. Receiver 1 never learns anything about the true state. If she observes *v*, however, she knows that receiver 2 knows the true state. If the sender would replace *v* by *w*, she would not learn anything at all.

This example illustrates that a receiver’s knowledge about the true state and the message profile realization can vary across signals, even if the latter induce identical distributions over posterior belief profiles. In particular, a receiver may have different knowledge about another receiver’s knowledge about the true state and the message profile realization. It is then natural to ask what types of signals that induce the same distribution over posteriors restrict this knowledge the most. In the example above, different messages might lead to the same posterior belief but to different higher order knowledge. By employing individually minimal or even language-independent signals we could avoid this issue. But even then: not all language-independent signals reveal the same higher order knowledge. To make this more precise, we define information correspondences that describe what receivers know about the state and the posterior belief profile (instead of the message profile realization), where we call a tuple of a state and a posterior belief profile a *posterior history*. A signal is more informative than another if for every receiver, every state, and every message profile that can occur in this state, the set of posterior histories that the receiver deems possible is smaller under the former than under the latter. We prove that for any inducible distribution over posterior belief profiles the least informative signals that induce it lie in the relative interior of the set of all language-independent signals that induce it.

The rest of the paper is organized as follows. In Sect. 2, we discuss related literature. In Sect. 3, we provide preliminary definitions and results. We then characterize sets of belief profiles that can be a subset of the support of an inducible distribution over posterior belief profiles in Sect. 4. In Sect. 5, we introduce minimal and individually minimal signals, and in Sect. 6, we turn to language-independent signals. In Sect. 7, we characterize inducible distributions of posteriors and provide several implications. Section 8 introduces information and posterior correspondences, and in Sect. 9, we explore the informativeness of signals.

## Related literature

Closely related to our analysis of inducible distributions of posteriors is Arieli et al. (2021). They consider multiple receivers who share a common prior belief on a binary state space and study joint posterior belief distributions. They first show that for the case of two receivers a quantitative version of the Agreement Theorem of Aumann (1976) holds; beliefs of receivers are approximately equal when they are approximately common knowledge. For more than two receivers, they relate the feasibility condition to the No Trade Theorem of Milgrom and Stokey (1982) and provide a characterization of feasible joint posteriors. These characterizations are then applied to study independent joint posterior belief distributions. While we pose the same question as Arieli et al. (2021), we obtain a completely different characterization while allowing for an arbitrary finite state space. Morris (2020) studies a similar problem as Arieli et al. (2021) and provides an alternative proof for one of their main results while delivering a many-state generalization. Another related paper is Ziegler (2020), who also provides a characterization of feasible joint posteriors. Arieli et al. (2021) show that the necessary and sufficient condition provided by Ziegler (2020) becomes insufficient if the support of a player’s posterior beliefs contains more than two points. In another related paper, He et al. (2022) consider the feasibility of distributions under private information structures and further analyze optimal communication under the constraint of not revealing any information about a variable correlated to the true state of the world.

Levy et al. (2021) also study the feasibility of joint distributions of posterior belief profiles and provide a necessary condition for which a distribution is feasible. They also show that the convex combination of a symmetric joint distribution and a fully correlated distribution with the same marginal distribution is inducible when the weight on the fully correlated distribution is sufficiently high. Finally, they demonstrate that a joint distribution satisfying their necessary condition becomes feasible when each belief profile in the support is moved sufficiently far in the direction of the prior.

There is literature in mathematics which studies the extent of difference in opinions of agents. Burdzy and Pal (2019) consider two experts who have access to different information and show that they can give radically different estimates of the probability of an event. In a related study, Burdzy and Pitman (2020) show that the opinion of two agents who share the same initial view can substantially differ if they have different sources of information, whereas Cichomski and Osekowski (2021) provide a bound for this difference in opinions. Related to these studies, we consider a sender who aims to drive a wedge between the beliefs of receivers by sending *correlated messages*. So, while they have the same source of information, their realizations are different from each other.

Like Ziegler (2020), Arieli et al. (2021), and Levy et al. (2021) we provide a characterization of inducible distributions over posterior belief profiles.^3^ Mathevet et al. (2020) focus instead on inducible distributions over belief hierarchies. Their characterization requires Bayes plausibility at the level of the sender and formulates two equations to obtain the correct belief hierarchies of the receivers. A central concept in their characterization is sender’s belief about the state given the entire profile of belief hierarchies. Our central tool is in terms of a matrix with dimensions given by the number of states and the number of posterior belief profiles.

While we focus on inducible distributions of posterior belief profiles, Bergemann and Morris (2016) consider a game-theoretic setup, study the distributions of receivers’ actions that the sender can induce, and characterize the set of Bayes correlated equilibria of the game. An advantage of their approach is that there is no need to make explicit use of information structures. They also develop an extension of the classic sufficiency condition of Blackwell (1953) for the multi-player setup and show that more information according to that criterion results in a smaller set of Bayes correlated equilibria. A similar setup is studied by Taneva (2019), who derives sender’s optimal information structure.

In the single-receiver case, introducing heterogeneity may render Bayes plausibility insufficient for a distribution to be inducible. Alonso and Camara (2016) consider a single receiver who does not share a common prior with the sender and show that an additional condition is required on top of Bayes plausibility. Beauchêne et al. (2019) also consider a single receiver, who is ambiguity averse, and a sender who may use an ambiguous communication device. In that case they are able to show that a modified version of Bayes plausibility holds. When there are multiple receivers, if information is perfectly correlated, then Bayes plausibility is still the only condition for inducibility since in this case all receivers have the same ex post belief. The first part of Wang (2013) and Alonso and Câmara (2016) both consider public communication and are examples of such a situation.

There is a wide literature that focuses on informativeness in the sense of Blackwell (1953).^4^ Rick (2013) considers an informed sender and an uninformed receiver and shows that miscommunication expands the set of distributions of beliefs the sender expects to induce. Gentzkow and Kamenica (2016) consider multiple senders and a single receiver and show that the amount of revealed information increases with the number of senders. Ichihashi (2019) considers a model of a single sender and receiver in which a designer can restrict the most informative message profile that the sender can generate, and he characterizes the information restriction that maximizes the receiver’s payoff. More recently, Brooks et al. (2022) and Mathevet and Taneva (2022) consider information hierarchies. While Brooks et al. (2022) show that one can always construct a collection of signals that induce Blackwell-ordered distributions such that the signals are refinement-ordered, Mathevet and Taneva (2022) also consider incentive compatibility of information transmission. While these papers compare the informativeness of different information structures by investigating the induced distributions of posteriors (or belief hierarchies), our analysis of informativeness follows the refinement tradition of Aumann (1976), where a source of information is represented as a partition of some extended state space. As is carefully explained in Green and Stokey (2022), both representations of information structures are equivalent, though the refinement tradition is more convenient when comparing information structures.

## Preliminaries and notation

Let $N=\left\{ 1,\ldots ,n\right\}$ be the set of receivers and let $\Omega$ be a finite set of *states* of the world. For any set *X*,  we denote by $\Delta (X)$ the set of probability distributions over *X* with finite support.^5^ We assume that sender and receivers share a common prior belief $\lambda ^0\in \Delta (\Omega )$.

Let $S_i$ be a non-empty set of *messages* sender can send to receiver $i\in N$, and let $S=\prod _{i\in N}S_i$. The elements of *S* are called *message profiles*.^6^ A *signal* is a function $\pi :\Omega \rightarrow \Delta \left( S\right)$ that maps each $\omega \in \Omega$ to a finite probability distribution over *S*. The set of possible message profile realizations is denoted by $S^{\pi }=\left\{ s\in S|\,\exists \omega \in \Omega :\pi (s|\omega )>0\right\} ,$ where $\pi (s\vert \omega )$ denotes the probability of message profile *s* conditional on the state being $\omega$. Note that $S^\pi$ is finite as $\pi \left( \cdot \vert \omega \right)$ has finite support for all $\omega \in \Omega$. Moreover, receiver $i \in N$ knows the joint distributions $\pi \left( \cdot \vert \omega \right)$ for all $\omega \in \Omega ,$ but only observes his private message $s_i$ when message profile *s* realizes. The set of all signals with message profiles in *S* is denoted by $\Pi (S).$ For each $\pi \in \Pi (S)$, $s_i\in S_i$, and $\omega \in \Omega$, let

$$\begin{aligned} \pi _i(s_i|\omega )=\sum _{t\in S: t_i=s_i}\pi (t|\omega ), \end{aligned}$$

which is the probability that receiver $i \in N$ observes $s_i$ given that the true state is $\omega$. (As $\pi (\cdot |\omega )$ has finite support, this is well defined.) For each $i \in N,$ define $S^{\pi }_i=\left\{ s_i\in S_i|\,\exists \omega \in \Omega :\pi _{i}(s_i|\omega )>0\right\}$, which is the (finite) set of messages receiver *i* observes with positive probability under $\pi$.

Given a signal $\pi \in \Pi (S)$, a message profile $s\in S^{\pi }$ generates the posterior belief profile $\lambda ^{\pi ,s}\in \Delta (\Omega )^n$ defined by

1 $$\begin{aligned} \lambda ^{\pi ,s}_i(\omega )=\frac{\pi _i(s_i|\omega )\lambda ^0(\omega )}{\sum _{\omega '\in \Omega }\pi _i(s_i|\omega ')\lambda ^0(\omega ')}, \quad \forall i \in N,~\forall \omega \in \Omega . \end{aligned}$$

So, $\lambda ^{\pi ,s}_i(\omega )$ is *i*’s posterior belief that the true state is $\omega$ upon receiving message $s_i$.

Recall that since $\Omega$ is finite and the sender employs finitely many messages conditional on the realization of $\omega \in \Omega$, the support of a signal is finite. A signal $\pi \in \Pi (S)$ *induces* the distribution $\sigma ^\pi$ over posterior belief profiles if for all $\lambda \in \Delta (\Omega )^n$ it holds that

2 $$\begin{aligned} \sigma ^\pi \left( \lambda \right) = \sum _{s\in S^{\pi }: \lambda ^{\pi ,s}=\lambda }\sum _{\omega \in \Omega }\pi (s|\omega )\lambda ^0(\omega ). \end{aligned}$$

In words, $\sigma ^\pi (\lambda )$ is the ex ante probability of posterior belief profile $\lambda$ given $\pi \in \Pi (S)$.^7^ By our assumptions made so far, the support of any inducible $\sigma$ is a finite set, i.e., $\sigma \in \Delta (\Delta (\Omega )^n)$. Given a set of message profiles *S*, we define the set of inducible distributions over posterior belief profiles by

$$\begin{aligned} \Sigma (S) = \left\{ \sigma \in \Delta (\Delta (\Omega )^n)\vert \,\exists \pi \in \Pi (S)\text { such that }\,\sigma ^{\pi } = \sigma \right\} . \end{aligned}$$

Observe that $\Sigma (S)$ depends on the set *S* of message profiles that the sender can use: a distribution $\sigma$ might only be inducible if *S* is sufficiently rich.^8^ This becomes relevant in situations where the sender’s message profile space is a priori limited, for example in case of schools who are bound to reveal information about students’ qualities within a grading framework (Boleslavsky & Cotton, 2015), or in case of a regulator who can reveal information about a bank’s financial situation only by a simple pass/fail stress test (Inostroza and Pavan, 2023). Thus, we will provide necessary and sufficient conditions on the size of *S* whenever appropriate.

We denote the support of $\sigma \in \Delta \left( \Delta \left( \Omega \right) ^n\right)$ by $\text {supp}(\sigma ).$ For each $i \in N$ and $\lambda _i \in \Delta (\Omega ),$ define

3 $$\begin{aligned} \sigma _i\left( \lambda _i\right) =\sum _{\lambda '\in \text {supp}(\sigma ):\lambda '_i=\lambda _i}\sigma (\lambda '). \end{aligned}$$

That is, $\sigma _i\left( \lambda _i\right)$ is the ex ante probability that receiver *i* will have posterior belief $\lambda _i$.

Let $\sigma , \sigma ^{\prime } \in \Delta (\Delta (\Omega )^{n})$ be two distributions over posterior belief profiles and let $\alpha \in [0,1].$ The convex combination $\hat{\sigma } = \alpha \sigma + (1-\alpha ) \sigma ^{\prime }$ is defined by

$$\begin{aligned} \hat{\sigma }(\lambda ) = \alpha \sigma (\lambda ) + (1-\alpha ) \sigma '(\lambda ), \quad \forall \lambda \in \Delta \left( \Omega \right) ^n. \end{aligned}$$

Even in the case with a single receiver, $\Sigma (S)$ need not be convex. For instance, if *S* consists of two messages, then it is possible to induce $\sigma , \sigma ^{\prime } \in \Sigma (S)$ with disjoint supports of cardinality 2. If $\hat{\sigma }$ is a strict convex combination of $\sigma$ and $\sigma ^{\prime }$, then $\left| \text {supp}\left( \hat{\sigma }\right) \right| =4$, so that $\hat{\sigma }$ cannot be induced with two messages only. The next result shows that $\Sigma (S)$ is convex when the message profile space is sufficiently rich.

### Proposition 3.1

Fix a set of message profiles *S* and let $\sigma ,\sigma '\in \Sigma (S)$ and $\alpha \in \left( 0,1\right)$. Then $\alpha \sigma + (1-\alpha ) \sigma ^{\prime } \in \Sigma (S)$ if and only if $\left| S_i\right| \ge \left| \rm{supp}(\sigma _i)\cup \rm{supp}(\sigma '_i)\right|$ for all $i\in N$.

### Proof

Let $$\hat{\sigma } = \alpha \sigma + (1-\alpha ) \sigma ^{\prime }$$.

If there is $i\in N$ such that $\left| S_i\right| < \left| \text {supp}(\sigma _i)\cup \text {supp}(\sigma '_i)\right|$, then there are not sufficient messages to implement all of *i*’s possible beliefs in $\text {supp}\left( \hat{\sigma }_i\right)$.

For the other direction, let $\left| S_i\right| \ge \left| \text {supp}(\sigma _i)\cup \text {supp}(\sigma '_i)\right|$ for all $i\in N$. Let $\pi , \pi ^{\prime } \in \Pi (S)$ be such that $\sigma ^{\pi } = \sigma$ and $\sigma ^{\pi '} = \sigma '$. Since $\left| S_i\right| \ge \left| \text {supp}(\sigma _i)\cup \text {supp}(\sigma '_i)\right|$, we can assume without loss of generality that there is $s\in S$ with $s_i\in S_i^{\pi }\cap S_i^{\pi '}$ if and only if there are $\lambda ^{\pi ,s}\in \text {supp}(\sigma )$ and $\lambda ^{\pi ',s}\in \text {supp}\left( \sigma '\right)$ such that $\lambda _i^{\pi ,s}=\lambda _i^{\pi ',s}$.

Let $\hat{\pi } = \alpha \pi + (1-\alpha ) \pi ^{\prime }$. Let $s \in S^{\hat{\pi }}$ and $i \in N$. Without loss of generality let $s_i \in S^{\pi }_{i}$. Assume first that $s_i\notin S_i^{\pi '}$. It holds that, for every $\omega \in \Omega$,

$$\begin{aligned} \lambda ^{\hat{\pi },s}_{i}(\omega ) = \frac{\hat{\pi }_i(s_i\vert \omega )\lambda ^0(\omega )}{\sum _{\omega '\in \Omega }\hat{\pi }_i(s_i\vert \omega ')\lambda ^0(\omega ')} =\frac{\alpha \pi _i(s_i\vert \omega )\lambda ^0(\omega )}{\alpha \sum _{\omega '\in \Omega }\pi _i(s_i\vert \omega ')\lambda ^0(\omega ')} =\lambda ^{\pi ,s}_i(\omega ). \end{aligned}$$

Assume next that $s_i\in S_i^{\pi '}$ and observe that in this case

$$\begin{aligned} \frac{\pi _i(s_i\vert \omega )\lambda ^0(\omega )}{\sum _{\omega '\in \Omega }\pi _i(s_i\vert \omega ')\lambda ^0(\omega ')} =\lambda _i^{\pi ,s}\left( \omega \right) = \lambda _i^{\pi ',s}\left( \omega \right) =\frac{\pi _i'(s_i\vert \omega )\lambda ^0(\omega )}{\sum _{\omega '\in \Omega }\pi _i'(s_i\vert \omega ')\lambda ^0(\omega ')} \end{aligned}$$

for all $\omega \in \Omega$. Thus,

$$\begin{aligned} \lambda ^{\hat{\pi },s}_{i}(\omega ) =\frac{\alpha \pi _i(s_i\vert \omega )\lambda ^0(\omega ) + \left( 1-\alpha \right) \pi _i'(s_i\vert \omega )\lambda ^0(\omega ) }{\alpha \sum _{\omega '\in \Omega }\pi _i(s_i\vert \omega ')\lambda ^0(\omega ')+\left( 1-\alpha \right) \sum _{\omega '\in \Omega }\pi _i'(s_i\vert \omega ')\lambda ^0(\omega ')} =\lambda ^{\pi ,s}_i(\omega ). \end{aligned}$$

We have shown that $\text {supp}\left( \hat{\sigma }\right) =\text {supp}(\sigma )\cup \text {supp}\left( \sigma '\right)$. We now have, for every $\lambda \in \Delta \left( \Omega \right) ^n,$

$$\begin{aligned} \hat{\sigma }(\lambda )&=\alpha \sigma (\lambda )+(1-\alpha )\sigma '(\lambda )\\&=\alpha \sum _{s\in S^{\pi }:\lambda ^{\pi ,s}=\lambda }\sum _{\omega \in \Omega }\pi (s\vert \omega )\lambda ^0(\omega )+(1-\alpha )\sum _{s\in S^{\pi '}:\lambda ^{\prime ^{s}}=\lambda }\sum _{\omega \in \Omega }\pi '(s\vert \omega )\lambda ^0(\omega ) \\&=\sum _{s\in S^{\hat{\pi }}:\hat{\lambda }^s=\lambda }\sum _{\omega \in \Omega }\hat{\pi }(s\vert \omega )\lambda ^0(\omega ). \end{aligned}$$

Hence, $$\hat{\pi }$$ induces $$\hat{\sigma }$$. $$\square$$

Most of the literature considers $S_i$ an arbitrary set that contains all messages that are necessary. The previous proposition implies that in this case the set of inducible posteriors is convex.

A distribution over posterior belief profiles $\sigma \in \Delta \left( \Delta \left( \Omega \right) ^n\right)$ is *Bayes-plausible* if

4 $$\begin{aligned} \sum _{\lambda _i\in \text {supp}(\sigma _i)}\lambda _i(\omega )\sigma _i\left( \lambda _i\right) =\lambda ^0(\omega ), \quad \forall i \in N,~\forall \omega \in \Omega . \end{aligned}$$

That is, for each receiver the expected posterior belief equals his prior belief. It is a well-known fact since Aumann et al. (1995) that Bayes plausibility (i.e., the martingale condition) is necessary and sufficient for inducibility when there is a single receiver, given that *S* is sufficiently rich. It now follows for the multiple receiver case that every $\sigma \in \Sigma (S)$ satisfies Bayes plausibility. We therefore obtain the following result, which is stated for later reference and without proof.

### Proposition 3.2

Let *S* be a set of message profiles. Every $\sigma \in \Sigma (S)$ is Bayes-plausible.

## Implementing belief profiles

When a sender is interacting with a single receiver who has no private information, Bayes plausibility of a distribution $\sigma \in \Delta (\Delta (\Omega ))$ is necessary and sufficient for $\sigma$ to belong to $\Sigma (S)$ when *S* is sufficiently rich. In particular, for any $\lambda \in \Delta (\Omega )$ there is $\sigma \in \Sigma (S)$ such that $\sigma (\lambda )>0$. In contrast, in the multiple receiver case it is not true that any single posterior belief profile $\lambda \in \Delta (\Omega )^n$ can occur with positive probability for a suitably chosen signal. Our first proposition shows that $\lambda \in \Delta (\Omega )^n$ can belong to the support of some $\sigma \in \Sigma (S)$ if and only if there is at least one state which, according to $\lambda$, is deemed possible by all receivers.

### Proposition 4.1

For every $i \in N,$ let $S_{i}$ contain at least two messages. Let $\lambda \in \Delta (\Omega )^n$. There exists $\sigma \in \Sigma (S)$ with $\sigma (\lambda )>0$ if and only if there is $\omega \in \Omega$ such that $\prod _{i\in N}\lambda _i(\omega )>0$.

### Proof

Assume $\pi \in \Pi (S)$ is such that $\sigma ^{\pi } = \sigma$ with $\sigma (\lambda ) > 0.$ Suppose that $\prod _{i\in N}\lambda _i(\omega )=0$ for all $\omega \in \Omega$, that is, for all $\omega \in \Omega$ there exists $i_{\omega } \in N$ such that $\lambda _{i_{\omega }}(\omega ) = 0.$ Let $s \in S^{\pi }$ be such that $\lambda ^{\pi ,s} = \lambda .$ Then it holds that, for all $\omega \in \Omega$, $\pi (s\vert \omega )\le \pi _{i_{\omega }}(s_{i_{\omega }} \vert \omega ) = 0.$ We find by (2) that $\sigma (\lambda )= 0,$ leading to a contradiction. Consequently, there exists $\omega \in \Omega$ such that $\prod _{i\in N}\lambda _i(\omega )>0$.

For the converse, assume there exists $\omega \in \Omega$ such that $\prod _{i\in N} \lambda _i(\omega )>0.$ For every $i\in N$ let $\beta _i=\max _{\omega \in \Omega }(\lambda _i(\omega )/\lambda ^0(\omega ))$ be the highest ratio across states of posterior belief to prior belief for receiver *i*. Let $x_{i},y_{i} \in S_{i}$ be two distinct messages. We define, for every $\omega \in \Omega ,$

$$\begin{aligned} \rho _i(x_{i}\vert \omega )&= \frac{1}{\beta _i} \frac{\lambda _i(\omega )}{\lambda ^0(\omega )},&\\ \rho _i(y_{i}\vert \omega )&=1-\rho _i(x_{i}\vert \omega ),&\\ \rho _{i}(s_{i}\vert \omega )&=0, \quad \forall s_{i} \in S_{i} \setminus \{x_{i}, y_{i}\}. \end{aligned}$$

Notice that $\rho _{i}(x_{i} \vert \omega ) \le 1.$ We define $\pi : \Omega \rightarrow \Delta \left( S\right)$ by

$$\begin{aligned} \pi (s \vert \omega ) = \prod _{i\in N} \rho _i(s_i \vert \omega ), \quad \forall s \in S,~\forall \omega \in \Omega . \end{aligned}$$

It holds that $\pi$ is a signal with $\pi _{i}(s_{i} \vert \omega ) = \rho _{i}(s_{i} \vert \omega )$ for every receiver $i \in N.$

Let $i \in N.$ For every $s \in S^{\pi }$ with $s_i=x_{i},$ for every $\omega \in \Omega ,$ it holds that

$$\begin{aligned} \lambda ^{\pi ,s}_i(\omega )=\frac{\pi _i(x_{i}\vert \omega )\lambda ^0(\omega )}{\sum _{\omega '\in \Omega }\pi _i(x_{i}\vert \omega ')\lambda ^0(\omega ')} =\frac{\frac{1}{\beta _i}\frac{\lambda _i(\omega )}{\lambda ^0(\omega )}\lambda ^0(\omega )}{\frac{1}{\beta _i}\sum _{\omega '\in \Omega }\frac{\lambda _i(\omega ')}{\lambda ^0(\omega ')}\lambda ^0(\omega ')} =\frac{\lambda _i(\omega )}{\sum _{\omega '\in \Omega }\lambda _i(\omega ')}=\lambda _i(\omega ). \end{aligned}$$

Thus, $$\lambda ^{\pi ,x} = \lambda ,$$ where $$x = (x_{1}, \ldots , x_{n})$$.

Let $\omega \in \Omega$ be such that $\lambda _i(\omega ) > 0$ for all $i\in N$. Then

$$\begin{aligned} \sigma \left( \lambda \right) \ge \pi \left( x\vert \omega \right) \lambda ^0\left( \omega \right) = \lambda ^0\left( \omega \right) \prod _{i\in N} \rho _i(x_i \vert \omega )>0, \end{aligned}$$

which implies that $\lambda \in \text {supp}(\sigma ^{\pi })$. $\square$

Note that we require $S_i$ to have at least two elements only for the “if” part of the proof. In the case $S_i$ consists of only one message, no information is provided and the posterior belief has to conform to the prior.

Let there be two receivers and a binary state space, say $\Omega =\left\{ X,Y\right\} ,$ as in our example in the introduction. It follows from Proposition 4.1 that a posterior belief profile $\lambda$ with $\lambda (X)=(0,1)$ cannot result with positive probability under *any* signal since $\lambda _1(X)\lambda _2(X)=0$ and $\lambda _1(Y)\lambda _2(Y)=0$. At the same time, for each $\varepsilon >0,$ the posterior belief profile $\lambda$ with $\lambda \left( X\right) =\left( \varepsilon ,1\right)$ can be obtained with positive probability.

Proposition 4.1 leads to some interesting comparisons with the Bayesian learning literature. Allon et al. (2021) consider a model in which agents obtain information about the true state of the world in a dynamic setting and study the evolution of beliefs. The authors show that even when the society’s prior belief distribution is not polarized, the prolonged learning process leads to a wedge between the agents’ beliefs; we show that the sender can create almost arbitrarily large disagreement between receivers by one-shot communication with positive probability, even when they have a common prior. In a related model, Mostagir and Siderius (2022) show that a planner who wishes to manipulate the beliefs of Bayesian agents in a particular way should target the agents who are the most polarized, i.e., whose beliefs are farthest away from the truth. Thus, if agents are initially in agreement, such a planner might benefit from first creating disagreement amongst the agents as described by Proposition 4.1 and then attempt to implement her preferred belief distribution.

We now generalize Proposition 4.1 from a single posterior belief profile to finite sets of posterior belief profiles.

### Proposition 4.2

Let $R\subseteq \Delta \left( \Omega \right) ^n$ be finite. For every $i \in N,$ let $S_{i}$ contain at least $\left| R_i\right| + 1$ messages, where $R_{i} = \text {proj}_i(R).$ There exists $\sigma \in \Sigma (S)$ with $R \subseteq \text {supp}(\sigma )$ if and only if for each $\lambda \in R$ there exists $\omega \in \Omega$ such that $\prod _{i\in N}\lambda _i(\omega )>0$.

### Proof

Proposition 4.1 implies necessity. For the other direction, let $R_i=\left\{ \lambda _i^1,\ldots ,\lambda _i^{m_i}\right\}$, let $\left\{ x_i^1,\ldots , x_i^{m_i}, y_i\right\} \subseteq S_i$ be such that $x^k_i\ne x_i^{\ell }, y_i$ for all $k\ne \ell$ and all $i\in N$. Let $R=\left\{ \lambda ^1,\ldots ,\lambda ^m\right\}$ and define $\pi ^1,\ldots ,\pi ^m$ as in the proof of Proposition 4.1, where, for all $i\in N$ and all $k = 1,\ldots ,m$ one has $\lambda ^{k} \in \text {supp}(\sigma ^{\pi ^{k}})$ and $S_i^{\pi ^k}\subseteq \left\{ x_i^k,y_i\right\}$. Let $\alpha ^1,\ldots ,\alpha ^m>0$ with $\sum _{k=1}^m\alpha ^k=1$, and let $\sigma =\sum _{k=1}^m\alpha ^k\sigma ^{\pi ^{k}}$. Since $\left| S_i\right| \ge m_i+1 = |\bigcup _{k=1}^m \text {supp}(\sigma _i^{\pi ^{k}})|$, iterative application of Proposition 3.1 implies that $\sigma \in \Sigma (S)$. Moreover, by construction, $\sigma ^{\pi }\left( \lambda ^k\right) =\alpha ^k\sigma ^{\pi ^{k}}\left( \lambda ^{k}\right) >0$. $\square$

Observe that Proposition 4.2 sharpens an earlier result in Sobel (2014). There the author showed that collections of strictly positive posterior belief profiles can be implemented. Our proposition characterizes the set of posterior belief profiles that can be implemented: in particular, we allow belief profiles that assign zero probability to some states as long as there is no such disagreement as in Proposition 4.1, i.e., as long as for each posterior belief profile there exists at least one state that is deemed possible by all receivers.

At this point we have identified sets that can be subsets of the support of an inducible distribution over posterior belief profiles. In Sect. 7 we characterize all inducible distributions over posterior belief profiles and the sets that can be the support of such distributions.

## Minimal and individually minimal signals

A large part of the literature is interested in “straightforward” signals (Kamenica & Gentzkow, 2011) that send recommendations to receivers about what action to take. In the present paper, we do not specify sets of feasible actions for receivers, so that sending recommendations has no meaning. Nevertheless, some signals are easier to handle than others and this and the next section will introduce some important classes.

Given a signal $\pi \in \Pi (S)$ and $s,s'\in S^{\pi }$ with $s\ne s'$, it is possible that $\lambda ^{\pi ,s}=\lambda ^{\pi ,s'}$. That is, two distinct message profiles can generate the same posterior belief profile. This motivates the following definition.

### Definition 5.1

Let *S* be a set of message profiles. A signal $\pi \in \Pi (S)$ is *minimal* if $\vert S^{\pi }\vert =\vert \text {supp}(\sigma ^{\pi })\vert$. The set of minimal signals is denoted by $\Pi ^{\mathrm m}(S)$.

Under a minimal signal, different message profiles lead to different posterior belief profiles. We give an illustration of a minimal signal in the following example.

### Example 5.2

Let $N=\left\{ 1,2\right\}$, $\Omega =\left\{ X,Y\right\}$, $S_1=\left\{ v,w\right\}$, and $S_2=\left\{ w,x,y\right\}$. Assume that agents have a common prior $\lambda ^0(X)=1/2$. Recall the $\pi$ in our illustrative example:

**Table:** 

$\pi$	(*v*, *x*)	(*v*, *y*)	(*w*, *w*)
*X*	$\frac{1}{2}$	0	$\frac{1}{2}$
*Y*	0	$\frac{1}{2}$	$\frac{1}{2}$

We have $S^{\pi }=\left\{ (v,x), (v,y), (w,w)\right\}$. Irrespective of the message received, receiver 1 gathers no information about the state: he has posterior beliefs $\lambda ^{\pi ,(v,x)}_1(X) = \lambda ^{\pi ,(v,y)}_1(X) = \lambda ^{\pi ,(w,w)}_1(X) = 1/2.$ For receiver 2, we have $\lambda ^{\pi ,(v,x)}_2(X)=1$, $\lambda ^{\pi ,(v,y)}_2(X)=0$, and $\lambda ^{\pi ,(w,w)}_2(X)=1/2$. It follows that

$$\begin{aligned} \text {supp}(\sigma ^{\pi })=\left\{ \left( (1/2,1/2), (1,0)\right) ,\left( (1/2,1/2), (0,1)\right) , \left( (1/2,1/2), (1/2,1/2)\right) \right\} . \end{aligned}$$

Since $\vert S^{\pi }\vert =\vert \text {supp}(\sigma ^{\pi })\vert$, $\pi$ is minimal. $\triangle$

In case of a single receiver, it is sufficient to have a bijection between $S^{\pi }$ and $\text {supp}(\sigma ^\pi )$ to ensure that each message leads to a different posterior, that is, to ensure that the signal employs a minimal number of messages. If there are multiple receivers, however, the existence of such a bijection does not guarantee that the number of messages for each receiver is indeed minimal. For instance, the two messages *v*, *w* in Example 5.2 both lead to the posterior belief $\lambda _{1}(X) = 1/2$ for receiver 1.

### Definition 5.3

Let *S* be a set of message profiles. A signal $\pi \in \Pi (S)$ is *i*-*minimal* if it holds that $\vert S^{\pi }_i\vert =\vert \text {supp}(\sigma ^{\pi }_i)\vert$. If $\pi$ is *i*-minimal for all $i\in N$, then we call $\pi$ *individually minimal*. The set of all individually minimal signals is denoted by $\Pi ^{\mathrm i}(S)$.

Under an individually minimal signal for each receiver any two different messages must lead to two different posterior beliefs. Hence, the number of different posterior beliefs a receiver can have equals the cardinality of $S^{\pi }_i$.

### Example 5.4

Recall the minimal signal $\pi$ in Example 5.2. Receiver 1 has the same posterior belief after observing *v* and observing *w*,  i.e., $\lambda ^{\pi ,(v,x)}_1(X)=\lambda ^{\pi ,(w,w)}_1(X)$. Thus, $\pi$ is not individually minimal. Consider the signal $\pi ^{\prime }$ defined by:

**Table:** 

$\pi ^{\prime }$	(*w*, *x*)	(*w*, *y*)	(*w*, *w*)
*X*	$\frac{1}{2}$	0	$\frac{1}{2}$
*Y*	0	$\frac{1}{2}$	$\frac{1}{2}$

We have $S^{\pi ^{\prime }}=\left\{ (w,x), (w,y), (w,w)\right\}$ and accordingly we can write the support of $\sigma ^{\pi ^{\prime }}$ as

$$\begin{aligned} \text {supp}(\sigma ^{\pi ^{\prime }})=\left\{ \left( (1/2,1/2), (1,0)\right) ,\left( (1/2,1/2), (0,1)\right) , \left( (1/2,1/2), (1/2,1/2)\right) \right\} . \end{aligned}$$

Note that $\text {supp}(\sigma ^{\pi })= \text {supp}(\sigma ^{\pi ^{\prime }})$. Since for all $s,t\in S^{\pi ^{\prime }}$ and each $i\in N$ we have $\lambda ^{\pi ',s}_i=\lambda ^{\pi ',t}_i$ if and only if $s_i=t_i$, $\pi ^{\prime }$ is individually minimal. $\triangle$

For any signal $\pi \in \Pi (S)$, $\vert S^{\pi }_i\vert =\vert \text {supp}(\sigma ^{\pi }_i)\vert$ for all $i\in N$ guarantees that a minimal number of messages is employed and implies that the number of employed message profiles is minimal as well. Thus, the following lemma does not come as a surprise.

### Lemma 5.5

Let *S* be a set of message profiles. It holds that $\Pi ^{\rm{i}}(S)\subseteq \Pi ^{\rm{m}}(S)$.

### Proof

Let $\pi \in \Pi ^{\mathrm i}(S)$. For each $i\in N$ there exists a bijection $\phi _i:S_i^{\pi }\rightarrow \text {supp}\left( \sigma ^{\pi }_i\right)$ since $\pi$ is individually minimal. In particular, for every $s \in S^{\pi },$ we have $\lambda ^{\pi ,s}=\left( \phi _i\left( s_{i}\right) \right) _{i\in N}$ so that there is a bijection between $S^{\pi }$ and $\text {supp}\left( \sigma ^{\pi }\right)$. Hence, $\vert S^{\pi }\vert =\vert \text {supp}(\sigma ^{\pi })\vert$, that is, $\pi$ is minimal. $\square$

We close this section by claiming that any distribution in $\Sigma (S)$ can be induced by an individually minimal signal. We do not provide a proof of Theorem 5.6 here, as it will follow easily from later results. The proof can be found after Corollary 7.3.

### Theorem 5.6

Let *S* be a set of message profiles. If $\sigma \in \Sigma (S),$ then there exists $\pi \in \Pi ^{\rm{i}}(S)$ such that $\sigma ^{\pi } = \sigma .$

## Language-independent signals

The same distribution over posterior belief profiles can be induced by various signals with potentially disjoint message profile spaces. We now proceed to show that there is a canonical way to describe signals. The principal idea is that the sender sends to each receiver the belief that he should have after observing the message.

### Definition 6.1

Let *S* be a set of message profiles. A signal $\pi \in \Pi (S)$ is a *language-independent signal* (LIS) if $S^{\pi } \subseteq \Delta (\Omega )^n$ and, for all $s \in S^{\pi },$ $\lambda ^{\pi ,s} = s.$ The set of language-independent signals is denoted by $\Pi ^{\ell }(S)$.

### Example 6.2

Let $N = \left\{ 1,2\right\}$, $\Omega =\left\{ X,Y\right\}$, and $\lambda ^0(X)=1/3$. The signal $\pi \in \Pi (S)$ is defined as follows:

**Table:** 

$\pi$	(*x*, *x*)	(*x*, *y*)	(*y*, *x*)	(*y*, *y*)
*X*	$\frac{1}{4}$	$\frac{1}{4}$	$\frac{1}{4}$	$\frac{1}{4}$
*Y*	$\frac{1}{8}$	$\frac{1}{8}$	$\frac{1}{8}$	$\frac{5}{8}$

For any $i\in N$, we have $\lambda ^{\pi ,(x,x)}_i(X)=1/2$ and $\lambda ^{\pi ,(y,y)}_i(X)=1/4$. Hence, $\pi$ is in fact individually minimal. The support of $\sigma ^{\pi }$ is equal to

$$\begin{aligned} \text {supp}(\sigma ^{\pi })&=\left\{ \lambda ^{\pi ,(x,x)},\lambda ^{\pi ,(x,y)},\lambda ^{\pi ,(y,x)},\lambda ^{\pi ,(y,y)}\right\} \\&=\left\{ \left(\left({\frac{1}{2}},{\frac{1}{2}}\right), \left({\frac{1}{2}},{\frac{1}{2}}\right)\right),\left(\left({\frac{1}{2}},{\frac{1}{2}}\right),\left({\frac{1}{4}},{\frac{3}{4}}\right)\right), \left(\left({\frac{1}{4}},{\frac{3}{4}}\right),\left({\frac{1}{2}},{\frac{1}{2}}\right)\right), \left(\left({\frac{1}{4}},{\frac{3}{4}}\right),\left({\frac{1}{4}},{\frac{3}{4}}\right)\right)\right\}. \end{aligned}$$

It holds that $\sigma ^{\pi }\left( \lambda ^{\pi ,(x,x)}\right) =\sigma ^{\pi }\left( \lambda ^{\pi ,(x,y)}\right) =\sigma ^{\pi }\left( \lambda ^{\pi ,(y,x)}\right) =1/6$ and $\sigma ^{\pi }\left( \lambda ^{\pi ,(y,y)}\right) =1/2$.

The signal $\pi ^{\prime } \in \Pi (S)$ is obtained by switching messages *x* and *y*,  so

**Table:** 

$\pi ^{\prime }$	(*x*, *x*)	(*x*, *y*)	(*y*, *x*)	(*y*, *y*)
*X*	$\frac{1}{4}$	$\frac{1}{4}$	$\frac{1}{4}$	$\frac{1}{4}$
*Y*	$\frac{5}{8}$	$\frac{1}{8}$	$\frac{1}{8}$	$\frac{1}{8}$

It is immediate that $\sigma ^{\pi } = \sigma ^{\pi ^{\prime }}.$

Next, consider the signal $\hat{\pi }$ that corresponds to the convex combination of $\pi$ and $\pi ^{\prime }$ with equal weights: $\hat{\pi } = 1/2 \pi + 1/2 \pi ^{\prime }.$ We have that

**Table:** 

$\hat{\pi }$	(*x*, *x*)	(*x*, *y*)	(*y*, *x*)	(*y*, *y*)
*X*	$\frac{1}{4}$	$\frac{1}{4}$	$\frac{1}{4}$	$\frac{1}{4}$
*Y*	$\frac{3}{8}$	$\frac{1}{8}$	$\frac{1}{8}$	$\frac{3}{8}$

Perhaps surprisingly, it holds that $\sigma ^{\hat{\pi }} \ne \sigma ^{\pi } = \sigma ^{\pi ^{\prime }}$.^9^ In fact, as can be verified easily, $\sigma ^{\hat{\pi }}$ is the distribution that assigns probability 1 to the posterior belief profile $\left( \lambda ^0,\lambda ^0\right)$. It follows that the set of signals which induce a particular distribution is not convex. Observe that $\hat{\pi }$ is not individually minimal, which implies that $\Pi^{\mathrm{i}}(S)$ is also not convex.

The signals $\pi ^{\ell },$ $\pi ^{\prime \ell },$ and $\hat{\pi }^{\ell }$ are obtained by relabeling the message profiles sent by $\pi ,$ $\pi ^{\prime },$ and $\hat{\pi },$ respectively, with the posterior belief profiles they lead to. We have that $\pi ^{\ell } = \pi ^{\prime \ell }.$ Both are equal to

**Table:** 

$\pi ^{\ell }, \pi ^{\prime \ell }$	$\left( (\frac{1}{2},\frac{1}{2}),(\frac{1}{2},\frac{1}{2})\right)$	$\left( (\frac{1}{2},\frac{1}{2}),(\frac{1}{4},\frac{3}{4})\right)$	$\left( (\frac{1}{4}, \frac{3}{4}),(\frac{1}{2},\frac{1}{2})\right)$	$\left( (\frac{1}{4},\frac{3}{4}),(\frac{1}{4},\frac{3}{4})\right)$
*X*	$\frac{1}{4}$	$\frac{1}{4}$	$\frac{1}{4}$	$\frac{1}{4}$
*Y*	$\frac{1}{8}$	$\frac{1}{8}$	$\frac{1}{8}$	$\frac{5}{8}$

Each receiver has posterior belief (1/2, 1/2) upon observing message (1/2, 1/2) and has posterior belief (1/4, 3/4) upon observing message (1/4, 3/4). Thus, $\pi ^{\ell }$ and $\pi ^{\prime \ell }$ are language-independent.

Finally, $\hat{\pi }^{\ell }$ sends $\lambda ^0$ to both players with probability 1. In particular, $\hat{\pi }^{\ell }$ is not a convex combination of $\pi ^{\ell }$ and $\pi '^{\ell }$. $\triangle$

The next result states that an LIS is individually minimal.

### Lemma 6.3

Let *S* be a set of message profiles. It holds that $\Pi ^{\mathrm \ell }(S) \subseteq \Pi ^{\mathrm i}(S).$

### Proof

Let $\pi \in \Pi ^{\ell }(S),$ $s \in S^{\pi },$ and $i \in N.$ It holds that $\lambda ^{\pi ,s}_{i} = s_{i}$ by definition of an LIS. This defines an identity between $S^{\pi }_{i}$ and $\text {supp}(\sigma ^{\pi }_{i}).$ It follows that $|S^{\pi }_{i}| = |\text {supp}(\sigma ^{\pi }_{i})|.$ $\square$

By Lemma 6.3 we know that an LIS is individually minimal and by Lemma 5.5 individual minimality implies minimality. Thus, there is a chain of inclusions between $\Pi ^{\ell }(S)$, $\Pi ^\mathrm{{i}}(S)$, and $\Pi ^\mathrm{{m}}(S)$.

### Corollary 6.4

Let *S* be a set of message profiles. Then $\Pi ^{\ell }(S)\subseteq \Pi ^\mathrm{{i}}(S)\subseteq \Pi ^\mathrm{{m}}(S)\subseteq \Pi (S)$.

Since we can transform any given individually minimal signal into an LIS by relabeling each message with the posterior belief that message leads to, an immediate consequence of Theorem 5.6 is that any element of $\Sigma (S)$ can be induced by an LIS if $\Delta (\Omega )^{n} \subseteq S,$ a result also obtained by Arieli et al. (2021) for a binary state space. We denote the set of all language-independent signals that induce a distribution $\sigma \in \Sigma (S)$ by $\Pi ^\ell (\sigma ).$ One advantage of language-independent signals is that for each $\sigma \in \Sigma (S)$ it holds that $\Pi ^\ell (\sigma )$ is convex. The proof of this statement, however, is postponed as it follows easily from later results, and can be found after Corollary 7.3.

### Proposition 6.5

Let *S* be a set of message profiles, $\Delta (\Omega )^n \subseteq S$, and $\sigma \in \Sigma (S).$ Then $\Pi ^{\ell }(\sigma )$ is non-empty and convex.

Proposition 6.5 contrasts Example 6.2 where we showed that both the set of all signals and the set of all individually minimal signals that induce a given $\sigma$ are typically not convex.^10^ This makes language-independent signals particularly attractive. Note that $\Pi ^\ell (\sigma )$ is not necessarily a singleton, as there might be different language-independent signals that induce a particular distribution of beliefs, which we illustrate later in Example 7.6. The multiplicity of elements of $\Pi ^\ell (\sigma )$ stems from the fact that language-independent signals might differ on the higher order beliefs even when they are inducing the same first-order beliefs.

**Equivalency of signals**

Recall that given an individually minimal signal, we can obtain an LIS by simply replacing messages with the posterior beliefs they lead to. More generally, given a signal $\pi \in \Pi (S)$, one can define $\pi '\in \Pi (S)$ by a one-to-one change in the names of messages in $S^{\pi }_i$ for each $i\in N$. In this case, we typically have $S^{\pi '}\ne S^{\pi }$, though we intuitively think of both signals as equivalent. More formally, we have the following definition.

### Definition 6.6

Let *S* and $\hat{S}$ be two sets of message profiles. Two signals $\pi : \Omega \rightarrow \Delta (S)$ and $\hat{\pi }: \Omega \rightarrow \Delta (\hat{S})$ are *equivalent* $(\pi \sim \hat{\pi })$ if for every $i \in N$ there is a bijection $\psi _{i}: S^{\pi }_{i} \rightarrow \hat{S}^{\hat{\pi }}_{i}$ such that, for every $\omega \in \Omega ,$ for every $s \in S^{\pi },$ $\hat{\pi }\left( \psi (s)\vert \omega \right) =\pi (s|\omega )$.^11^

We can interpret equivalent signals as providing the same information in different languages. Indeed, let $s_{i} \in S^{\pi }_{i}$ and $\hat{s}_{i}\in \hat{S}^{\hat{\pi }}_{i}$ be such that $\psi _{i}(s_{i}) = \hat{s}_{i}.$ It holds that

$$\begin{aligned} \pi _i(s_i\vert \omega )=\sum _{t\in S^{\pi }:t_i=s_i}\pi (t\vert \omega )=\sum _{t\in S^{\pi }:t_i=s_i} \hat{\pi }\left( \psi (t)\vert \omega \right) =\sum _{\hat{t} \in \hat{S}^{\hat{\pi }}: \hat{t}_i = \hat{s}_i} \hat{\pi }(\hat{t} \vert \omega ) = \hat{\pi }_i(\hat{s}_i\vert \omega ), \quad \forall \omega \in \Omega . \end{aligned}$$

Now consider $s \in S^{\pi }$ and $\hat{s} \in \hat{S}^{\hat{\pi }}$ such that $\hat{s} = \psi (s).$ For every $i \in N,$ we have that

5 $$\begin{aligned} \lambda ^{\pi ,s}_{i}(\omega ) = \frac{\pi _{i}(s_{i}| \omega ) \lambda ^{0}(\omega )}{\sum _{\omega ^{\prime } \in \Omega } \pi _{i}(s_{i} | \omega ^{\prime }) \lambda ^{0}(\omega ^{\prime })} = \frac{\hat{\pi }_{i}(\hat{s}_{i}| \omega ) \lambda ^{0}(\omega )}{\sum _{\omega ^{\prime } \in \Omega } \hat{\pi }_{i}(\hat{s}_{i} | \omega ^{\prime }) \lambda ^{0}(\omega ^{\prime })} = \lambda ^{\hat{\pi },s}_{i}(\omega ), \quad \forall \omega \in \Omega . \end{aligned}$$

It follows from (5) that sending message profile *s* under signal $\pi$ and sending message profile $\hat{s}$ under signal $\hat{\pi }$ results in the same posterior belief profile. It is also immediate from Definition 6.6 that $\hat{S}^{\hat{\pi }} = \psi (S^{\pi }).$

The next proposition, stating that equivalent signals induce the same distribution over posterior belief profiles, now follows easily.

### Proposition 6.7

Fix two sets of message profiles *S* and $\hat{S}$, and let $\pi : \Omega \rightarrow \Delta (S)$ and $\hat{\pi }: \Omega \rightarrow \Delta (\hat{S})$ be such that $\pi \sim \hat{\pi }.$ It holds that $\sigma ^{\pi } = \sigma ^{\hat{\pi }}.$

### Proof

For every $i \in N$ there is a bijection $\psi _{i}: S^{\pi }_{i} \rightarrow \hat{S}^{\hat{\pi }}_{i}$ such that, for every $\omega \in \Omega ,$ for every $s \in S^{\pi },$ $\hat{\pi }\left( \psi (s)\vert \omega \right) =\pi (s|\omega )$. Let $s\in S^{\pi }$ and $\hat{s} \in \hat{S}^{\hat{\pi }}$ be such that $\psi (s) = \hat{s}.$ It follows from (5) that $\lambda ^{\pi ,s} = \lambda ^{\hat{\pi },\hat{s}}.$ Since $\hat{S}^{\hat{\pi }} = \psi (S^{\pi }),$ we have that $\text {supp}(\sigma ^{\hat{\pi }})= \text {supp}(\sigma ^{\pi }).$ Moreover, it holds that, for every $\lambda \in \text {supp}(\sigma ^{\pi }),$

$$\begin{aligned} \sigma ^{\pi }\left( \lambda \right)&=\sum _{s\in S^{\pi }:\lambda ^{\pi ,s}=\lambda }\sum _{\omega \in \Omega }\pi (s\vert \omega )\lambda ^0(\omega ) =\sum _{s\in S^{\pi }:\lambda ^{\pi ,s}=\lambda }\sum _{\omega \in \Omega }\hat{\pi }\left( \psi (s)\vert \omega \right) \lambda ^0(\omega ) \\&=\sum _{\hat{s} \in \hat{S}^{\hat{\pi }} : \lambda ^{\hat{\pi },\hat{s}} = \lambda }\sum _{\omega \in \Omega } \hat{\pi }(\hat{s}\vert \omega ) \lambda ^0(\omega ) =\sigma ^{\hat{\pi }}\left( \lambda \right) . \end{aligned}$$

$$\square$$

Note that the converse of Proposition 6.7 is not true: as we will see in Example 7.6 there are signals that induce the same distribution over posterior belief profiles but that are not equivalent.

The next proposition shows that each set of equivalent signals contains at most one LIS.

### Proposition 6.8

Fix a set of message profiles *S* and let $\pi ,\pi '\in \Pi ^\ell (S)$ with $\pi \sim \pi '$. It holds that $\pi =\pi ^{\prime }$.

### Proof

By Proposition 6.7 it holds that $\sigma ^{\pi } = \sigma ^{\pi ^{\prime }},$ so $S^{\pi }= \text {supp}\left( \sigma ^{\pi }\right) = \text {supp}\left( \sigma ^{\pi ^{\prime }}\right) = S^{\pi '}.$ As $\pi \sim \pi ',$ for every $i \in N$ there is a bijection $\psi _{i}: S^{\pi }_{i} \rightarrow S^{\pi '}_{i}$ such that, for every $\omega \in \Omega ,$ for every $s \in S^{\pi },$ $\pi '\left( \psi (s)\vert \omega \right) =\pi (s|\omega )$. In particular, since $\pi , \pi ' \in \Pi ^{\ell }(S),$ we have, for every $i \in N,$ for every $\lambda \in S^{\pi },$

6 $$\begin{aligned} \psi _i\left( \lambda _{i}\right) \left( \omega \right) = \frac{\pi _i'\left( \psi _i\left( \lambda _{i}\right) \vert \omega \right) \lambda ^0\left( \omega \right) }{\sum _{\omega '\in \Omega }\pi _i'\left( \psi _i\left( \lambda _{i}\right) \vert \omega '\right) \lambda ^0\left( \omega '\right) } =\frac{\pi _i\left( \lambda _{i}\vert \omega \right) \lambda ^0\left( \omega \right) }{\sum _{\omega '\in \Omega }\pi _i\left( \lambda _{i}\vert \omega '\right) \lambda ^0\left( \omega ^{\prime }\right) } =\lambda _i\left( \omega \right) , \quad \forall \omega \in \Omega , \end{aligned}$$

where the first and third equality follow since $\pi , \pi ^{\prime } \in \Pi ^{\ell }(S)$, and the second equality uses (5). It follows that $\pi =\pi ^{\prime }$. $\square$

Observe that a signal that is not individually minimal cannot be equivalent to an LIS as the required bijection between message spaces cannot exist. Nevertheless for every signal there is a canonical way to find an LIS that induces the same posterior. The construction heavily relies on the following lemma, which is straightforward and, therefore, stated without proof.^12^

### Lemma 6.9

Fix a set of message profiles *S* and let $\pi \in \Pi (S)$ be a signal. It holds that

$$\begin{aligned} \frac{\sum _{s_i\in S_i^{\pi }:\lambda _i^{\pi ,s}=\lambda _i}\pi _i\left( s_i|\omega \right) \lambda ^0\left( \omega \right) }{\sum _{\omega ^{\prime }\in \Omega }\sum _{s_i\in S_i^{\pi }:\lambda _i^{\pi ,s}=\lambda _i}\pi _i\left( s_i|\omega ^{\prime }\right) \lambda ^0\left( \omega ^{\prime }\right) }=\lambda _i\left( \omega \right) , \quad \forall \omega \in \Omega ,~\forall i \in N,~\forall \lambda _{i} \in \rm{supp}\left( \sigma ^{\pi }_{i}\right) . \end{aligned}$$

Lemma 6.9 extends the formula for Bayesian updating and applies it to all messages simultaneously that lead to a particular posterior belief. According to the lemma, distinct messages that lead to the same posterior can be replaced by the same message. Thus, the following result is immediate and we present it without proof.

### Corollary 6.10

Fix a set of message profiles *S* and let $\Delta (\Omega )^{n} \subseteq S$. For $\pi \in \Pi (S)$ define $\pi ^{\ell }: \Omega \rightarrow \Delta (S)$ as

7 $$\begin{aligned} \pi ^{\ell } \left( \lambda \vert \omega \right) = \sum _{s\in S^{\pi }:\lambda ^{\pi ,s}=\lambda } \pi \left( s\vert \omega \right) , \quad \forall \omega \in \Omega ,~\forall \lambda \in \rm{supp}\left( \sigma ^{\pi }\right) . \end{aligned}$$

Then $\sigma ^{\pi ^{\ell }}=\sigma ^{\pi }$. Moreover, if $\pi \in \Pi ^\mathrm{{i}}(S)$ then $\pi ^{\ell }\sim \pi$.

## Inducible distributions

Unlike in the single-receiver case, when dealing with multiple receivers, Bayes plausibility alone is not sufficient to ensure that a distribution over posterior belief profiles belongs to $\Sigma (S).$^13^

### Example 7.1

Let $N=\left\{ 1,2,3\right\}$, $\Omega =\left\{ X,Y\right\}$, and $S = \Delta (\Omega )^{3}.$ Assume the agents have common prior $\lambda ^0(X)=1/6$. Let $\lambda ^1(X)=(1/2,1/2,0)$, $\lambda ^2(X)=(1/2,0,1/2)$, $\lambda ^3(X)=(0,1/2,1/2),$ and $\lambda ^4(X) = (0,0,0)$ and let $\sigma \in \Delta \left( \Delta \left( \Omega \right) ^3\right)$ be given by $\sigma \left( \lambda ^1\right) =\sigma \left( \lambda ^2\right) =\sigma \left( \lambda ^3\right) =1/6$ and $\sigma \left( \lambda ^4\right) =1/2$. Then, for each $i\in N,$ we have $\sigma _i \left( 1/2,1/2\right) = 1/3$ and $\sigma _i(0,1) = 2/3.$

First note that $\sigma$ is Bayes-plausible:

$$\begin{aligned} \sum _{\lambda _i\in \text {supp}(\sigma _i)}\lambda _i(X)\sigma _i\left( \lambda _i\right) =\frac{1}{2}\cdot \sigma _i(1/2,1/2)+0\cdot \sigma _i(0,1)=\frac{1}{2}\cdot \frac{1}{3}=\frac{1}{6}=\lambda ^0(X), \quad \forall i \in N. \end{aligned}$$

Suppose that signal $\pi \in \Pi (S)$ induces $\sigma .$ By Corollary 6.10 it is without loss of generality to assume that $\pi \in \Pi ^{\ell }(S).$ In this case, for any receiver, observing (1/2,1/2) leads to posterior belief (1/2,1/2), and observing (0,1) leads to posterior belief (0,1). This implies that no receivers can observe (0,1) in state *X*, i.e., $\pi _i((0,1)|X)=0$ for all $i\in N$. It follows that $\pi \left( \lambda ^1|X\right) =\pi \left( \lambda ^2|X\right) =\pi \left( \lambda ^3|X\right) =\pi \left( \lambda ^4|X\right) =0$, which obviously leads to a contradiction. $\triangle$

To guarantee that a distribution over posterior belief profiles belongs to $\Sigma (S),$ additional conditions need to be imposed on top of Bayes plausibility. In Theorem 7.2, we provide necessary and sufficient conditions for a distribution over posterior belief profiles to belong to $\Sigma (S)$.

### Theorem 7.2

Let *S* be a set of message profiles and $\sigma \in \Delta (\Delta (\Omega )^n)$ be such that, for every $i \in N,$ $|S_{i}| \ge |\rm{supp}(\sigma _{i})|.$ Then $\sigma \in \Sigma (S)$ if and only if $\sigma$ is Bayes-plausible and there exists $p \in \mathbb R^{\Omega \times \text {supp}(\sigma )}_+$ such that

$$\begin{aligned} \begin{array}{lll} (i) &{} \sum _{\omega \in \Omega }p(\omega ,\lambda )=\sigma \left( \lambda \right) , & \forall \lambda \in \rm{supp}(\sigma ), \\ (ii) &{} \sum _{\lambda ^{\prime } \in \text {supp}(\sigma ):\lambda ^{\prime }_i=\lambda _i}p(\omega ,\lambda ^{\prime })=\lambda _i(\omega )\sigma _i\left( \lambda _i\right) , &{} \forall \omega \in \Omega ,~\forall i \in N,~\forall \lambda _i\in \rm{supp}(\sigma _i). \end{array} \end{aligned}$$

If $\sigma \in \Sigma (S),$ then the signal $\pi :\Omega \rightarrow \Delta (\Delta (\Omega )^n)$ defined by

8 $$\begin{aligned} \pi \left( \lambda \vert \omega \right) =\frac{p(\omega ,\lambda )}{\lambda ^0(\omega )}, \quad \forall \omega \in \Omega ,~\forall \lambda \in \rm{supp}(\sigma ), \end{aligned}$$

is an LIS such that $\sigma ^{\pi } = \sigma .$

Condition (*i*) can be interpreted as “posterior marginality" as it states that the probability of a posterior belief profile $\lambda$ is the marginal of $p(\omega ,\lambda )$. The right-hand side of condition (*ii*) is the probability that $\omega$ is the true state according to *i*’s belief $\lambda _i$ multiplied with the probability that *i* has belief $\lambda _i$. Thus, the sum on the left-hand side is the probability that *i* has belief $\lambda _i$ *and* $\omega$ is the true state. Intuitively, $p(\omega ,\lambda )$ can be interpreted as the probability of the state being $\omega$ and the induced posterior being $\lambda$.

### Proof

Assume that $\sigma$ is Bayes-plausible and there exists $p \in \mathbb R^{\Omega \times \text {supp}(\sigma )}_+$ such that (*i*) and (*ii*) are satisfied. Let $\pi$ be defined as in (8). We first show that $\pi$ is a signal.

Let $\omega \in \Omega .$ Obviously, it holds that, for every $\lambda \in \Delta (\Omega )^{n},$ $\pi (\lambda | \omega ) \ge 0.$ In formula (9) that follows next, $i \in N$ is an arbitrarily chosen receiver. It holds that

9 $$\begin{aligned} \sum _{\lambda \in S^{\pi }}p\left( \omega ,\lambda \right) =\sum _{\lambda _i\in \text {supp}\left( \sigma _i\right) }\sum _{\lambda '\in \text {supp}(\sigma ):\lambda '_i=\lambda _i}p(\omega ,\lambda ') {\mathop {=}\limits ^{(ii)}}\sum _{\lambda _i\in \text {supp}\left( \sigma _i\right) }\lambda _i(\omega )\sigma _i\left( \lambda _i\right) =\lambda ^0(\omega ), \end{aligned}$$

where the last equality is true as $\sigma$ is Bayes-plausible. We find that

$$\begin{aligned} \sum _{\lambda \in S^{\pi }}\pi (\lambda \vert \omega ) = \sum _{\lambda \in S^{\pi }} \frac{p\left( \omega ,\lambda \right) }{\lambda ^0(\omega )}{\mathop {=}\limits ^{(9)}}\frac{\lambda ^0(\omega )}{\lambda ^0(\omega )}=1, \end{aligned}$$

which proves that $\pi$ is a signal.

Next, we show that $\pi$ is an LIS. Let $\omega \in \Omega ,$ $i \in N,$ and $\lambda _i \in \text {supp}(\sigma _{i}).$ It holds that

$$\begin{aligned} \frac{\pi _i(\lambda _i|\omega )\lambda ^0(\omega )}{\sum _{\omega '\in \Omega }\pi _i(\lambda _i|\omega ')\lambda ^0(\omega ')}&= \frac{\sum _{\lambda ^{\prime }\in \text {supp}(\sigma ):\lambda ^{\prime }_i=\lambda _i}\pi (\lambda ^{\prime }|\omega )\lambda ^0(\omega )}{\sum _{\omega '\in \Omega }\sum _{\lambda ^{\prime }\in \text {supp}(\sigma ):\lambda ^{\prime }_i=\lambda _i}\pi (\lambda ^{\prime }|\omega ')\lambda ^0(\omega ')} \\&{\mathop {=}\limits ^{(8)}}\frac{\sum _{\lambda ^{\prime } \in \text {supp}(\sigma ):\lambda ^{\prime }_i=\lambda _i}\frac{p\left( \omega ,\lambda ^{\prime }\right) }{\lambda ^0(\omega )}\lambda ^0(\omega )}{\sum _{\omega '\in \Omega }\sum _{\lambda ^{\prime }\in \text {supp}(\sigma ):\lambda ^{\prime }_i=\lambda _i}\frac{p\left( \omega ',\lambda ^{\prime }\right) }{\lambda ^0(\omega ')}\lambda ^0(\omega ')} \\&=\frac{\sum _{\lambda ^{\prime }\in \text {supp}(\sigma ):\lambda ^{\prime }_i=\lambda _i}p\left( \omega ,\lambda ^{\prime }\right) }{\sum _{\omega '\in \Omega }\sum _{\lambda ^{\prime }\in \text {supp}(\sigma ):\lambda ^{\prime }_i=\lambda _i}p\left( \omega ',\lambda ^{\prime }\right) } \\&{\mathop {=}\limits ^{(ii)}}\frac{\lambda _i(\omega )\sigma _i\left( \lambda _i\right) }{\sum _{\lambda ^{\prime }\in \text {supp}(\sigma ):\lambda ^{\prime }_i=\lambda _i} \sum _{\omega ^{\prime } \in \Omega } p\left( \omega ',\lambda ^{\prime }\right) } \\&{\mathop {=}\limits ^{(i)}}\frac{\lambda _i(\omega )\sigma _i\left( \lambda _i\right) }{\sum _{\lambda ^{\prime }\in \text {supp}(\sigma ):\lambda ^{\prime }_i=\lambda _i}\sigma \left( \lambda ^{ \prime }\right) } \\&=\frac{\lambda _i(\omega )\sigma _i\left( \lambda _i\right) }{\sigma _i\left( \lambda _i\right) } \\&=\lambda _i(\omega ). \end{aligned}$$

As message $\lambda _i$ leads to posterior $\lambda _i$, $\pi$ is an LIS.

We show next that $\sigma ^{\pi } = \sigma .$ If $\sigma \left( \lambda \right) =0$, then $\sigma ^{\pi }\left( \lambda \right) =0$ by construction. So, let $\lambda \in \text {supp}(\sigma )$. It holds that

$$\begin{aligned} \sigma ^{\pi }(\lambda ) = \sum _{\omega \in \Omega }\pi (\lambda |\omega )\lambda ^0(\omega ) =\sum _{\omega \in \Omega }\frac{p\left( \omega ,\lambda \right) }{\lambda ^0(\omega )}\lambda ^0(\omega ) =\sum _{\omega \in \Omega }p\left( \omega ,\lambda \right) {\mathop {=}\limits ^{(i)}}\sigma (\lambda ). \end{aligned}$$

At this point we have shown that $\sigma$ is inducible if $\text {supp}(\sigma _i)\subseteq S_i$. Recall that $\left| S_i\right| \ge \text {supp}(\sigma _i)$. For every $i \in N,$ let $T_{i}$ be a subset of $S_{i}$ with cardinality equal to $\left| \text {supp}(\sigma _{i})\right|$ and take a bijection $\psi _{i}: \text {supp}(\sigma _{i}) \rightarrow T_{i}.$ Define the signal $\pi ^{\prime }: \Omega \rightarrow \Delta (S)$ by

$$\begin{aligned} \pi ^{\prime }(\psi (\lambda ) | \omega ) = \pi (\lambda | \omega ), \quad \forall \omega \in \Omega ,~\forall \lambda \in \text {supp}(\sigma ). \end{aligned}$$

Then $\pi \sim \pi ^{\prime }$, so by Proposition 6.7 we have that $\sigma ^{\pi ^{\prime }} = \sigma ^{\pi } = \sigma$. It follows that $\sigma \in \Sigma (S).$

Now assume that $\sigma \in \Sigma (S).$ It follows from Proposition 3.2 that $\sigma$ is Bayes-plausible. Let $\pi \in \Pi (S)$ be such that $\sigma ^{\pi } = \sigma .$ For every $\omega \in \Omega ,$ for every $\lambda \in \text {supp}(\sigma )$, define

10 $$\begin{aligned} p\left( \omega ,\lambda \right) =\sum _{s\in S^{\pi }:\lambda ^{\pi ,s}=\lambda }\pi (s|\omega )\lambda ^0(\omega ). \end{aligned}$$

We first show that (*i*) holds. We have that

$$\begin{aligned} \sigma \left( \lambda \right) =\sum _{s\in S^{\pi }:\lambda ^{\pi ,s}=\lambda }\sum _{\omega \in \Omega }\pi (s|\omega )\lambda ^0(\omega )&{\mathop {=}\limits ^{(10)}}\sum _{\omega \in \Omega }p\left( \omega ,\lambda \right) , \quad \forall \lambda \in \text {supp}(\sigma ). \end{aligned}$$

Next, we show (*ii*) holds. Let $\omega \in \Omega ,$ $i\in N,$ and $\lambda _i \in \text {supp}(\sigma _i).$ We have that

$$\begin{aligned} \lambda _i(\omega )\sigma _i\left( \lambda _i\right)&=\frac{\sum _{s_i\in S_i^{\pi }:\lambda _i^{\pi ,s}=\lambda _i}\pi _i(s_i|\omega )\lambda ^0(\omega )}{\sum _{\omega ^{\prime }\in \Omega }\sum _{s_i\in S_i^{\pi }:\lambda _i^{\pi ,s}=\lambda _i}\pi _i(s_i|\omega ^{\prime })\lambda ^0(\omega ^{\prime })}\sum _{\lambda ^{\prime }\in \text {supp}(\sigma ):\lambda ^{\prime }_i=\lambda _i}\sigma \left( \lambda ^{\prime }\right) \\&=\frac{\sum _{s_i\in S_i^{\pi }:\lambda _i^{\pi ,s}=\lambda _i}\pi _i(s_i|\omega )\lambda ^0(\omega )}{\sum _{\omega ^{\prime }\in \Omega }\sum _{s_i\in S_i^{\pi }:\lambda _i^{\pi ,s}=\lambda _i}\pi _i(s_i|\omega ^{\prime })\lambda ^0(\omega ^{\prime })}\sum _{\lambda ^{\prime }\in \text {supp}(\sigma ):\lambda ^{ \prime }_i=\lambda _i}\sum _{s\in S^{\pi }:\lambda ^{\pi ,s}=\lambda ^{\prime }}\sum _{\omega ^{\prime }\in \Omega }\pi \left( s|\omega ^{\prime }\right) \lambda ^0\left( \omega ^{\prime }\right) \\&=\frac{\sum _{s_i\in S_i^{\pi }:\lambda _i^{\pi ,s}=\lambda _i}\pi _i(s_i|\omega )\lambda ^0(\omega )}{\sum _{\omega ^{\prime }\in \Omega }\sum _{s_i\in S_i^{\pi }:\lambda _i^{\pi ,s}=\lambda _i}\pi _i(s_i|\omega ^{\prime })\lambda ^0(\omega ^{\prime })}\sum _{\omega ^{\prime }\in \Omega }\sum _{s_i\in S_i^{\pi }:\lambda _i^{\pi ,s}=\lambda _i}\pi _i\left( s_i|\omega ^{\prime }\right) \lambda ^0\left( \omega ^{\prime }\right) \\&=\sum _{s_i\in S_i^{\pi }:\lambda _i^{\pi ,s}=\lambda _i}\pi _i(s_i|\omega )\lambda ^0(\omega )\\&=\sum _{\lambda ^{\prime }\in \text {supp}(\sigma ):\lambda ^{\prime }_i=\lambda _i}\sum _{s\in S^{\pi }:\lambda ^{\pi ,s}=\lambda ^{\prime }}\pi \left( s|\omega \right) \lambda ^0\left( \omega \right) \\&=\sum _{\lambda ^{\prime } \in \text {supp}(\sigma ):\lambda ^{\prime }_i=\lambda _i}p\left( \omega ,\lambda ^{\prime }\right) , \end{aligned}$$

where the first equality follows from Lemma 6.9. $\square$

Theorem 7.2 explicitly shows what is needed in addition to Bayes plausibility to ensure that a distribution over posterior belief profiles belongs to $\Sigma (S).$ Observe that any $p \in \mathbb R^{\Omega \times \text {supp}(\sigma )}_+$ which satisfies Condition (*i*) is a finite probability distribution, that is, $p\in \Delta \left( \Omega \times \text {supp}(\sigma )\right)$.^14^

Note that while we pose a similar question to Arieli et al. (2021) and Ziegler (2020), we obtain a completely different characterization. To obtain a characterization for more than two players and a binary state space, Arieli et al. (2021) utilize the No Trade Theorem of Milgrom and Stokey (1982). After introducing a mediator who trades with the agents they provide an interval for the mediator’s expected payoff for a distribution to be inducible.^15^ Ziegler (2020) generalizes Kamenica and Gentzkow (2011) to two players and makes use of “belief-dependence bounds” to provide a characterization for inducible distributions, which are defined over the CDFs associated with distributions of beliefs. In contrast, we allow for both a general finite state space *and* a finite number of receivers, and provide a characterization of inducible posteriors in terms of a system of equations, which represents the properties of the agents’ marginal beliefs.

Observe that by Equations (8) and (9) *p* is a common prior over $\Omega \times \text {supp}(\sigma )$. Thus, Theorem 7.2 bears some resemblance to Proposition 1 in Mathevet et al. (2020). Yet, while they impose conditions on the common prior over belief hierarchies from which the posterior distribution emerges, our condition is formulated as a system of separate marginality conditions for all players.

While Theorem 7.2 is useful in determining whether a distribution of beliefs is inducible, it also provides an LIS that induces the desired distribution. In Example 7.4, we use Theorem 7.2 to show that a given distribution of beliefs is not inducible. In Example 7.6, we use two solutions to conditions (*i*) and (*ii*) to provide two distinct LIS’s that induce the same distribution. Yet, beforehand, we provide the proofs that have been left out in Sects. 5 and 6.

For any $\sigma \in \Sigma (S),$ define

$$\begin{aligned} P(\sigma )=\left\{ p \in \mathbb {R}^{\Omega \times \text {supp}(\sigma )}_{+} \vert \, p\text { satisfies (i) and (ii) of Theorem 7.2}\right\} . \end{aligned}$$

As $P(\sigma )$ is defined as the set of non-negative matrix solutions to a system of linear equalities, where the system is such that the components of any solution matrix sum up to one, we immediately have the following result.

### Corollary 7.3

Let *S* be a set of message profiles. For every $\sigma \in \ \Sigma (S),$ $P(\sigma )$ is a non-empty, compact, and convex polytope.

We are now ready to provide the remaining proofs of Sects. 5 and 6.

### Proof of Theorem 5.6

Let $\sigma \in \Sigma (S)$. Then it holds that, for every $i \in N,$ $|S_{i}| \ge \text {supp}(\sigma _{i})$. Theorem 7.2 implies that there is an LIS $\pi : \Omega \rightarrow \Delta (\Delta (\Omega )^{n})$ which induces $\sigma .$ For every $i \in N,$ let $T_{i}$ be a subset of $S_{i}$ with cardinality equal to $|\text {supp}(\sigma _{i})|$ and take a bijection $\psi _{i}: \text {supp}(\sigma _{i}) \rightarrow T_{i}$. Let the signal $\pi ^{\prime }: \Omega \rightarrow \Delta (S)$ be defined by

$$\begin{aligned} \pi ^{\prime }(\psi (\lambda ) | \omega ) = \pi (\lambda | \omega ), \quad \forall \omega \in \Omega ,~\lambda \in \text {supp}(\sigma ). \end{aligned}$$

Then $\pi \sim \pi ^{\prime },$ so by Proposition 6.7 we have that $\sigma ^{\pi ^{\prime }} = \sigma ^{\pi } = \sigma$. As the LIS $\pi$ is individually minimal, it follows that $\pi ^{\prime } \in \Pi^{\mathrm{i}}(S).$ $\square$

### Proof of Proposition 6.5

As $P(\sigma )$ is a non-empty, compact, and convex polytope by Corollary 7.3 and $\Pi ^\ell \left( \sigma \right)$ is a linear transformation of $P(\sigma )$ by (8), $\Pi ^\ell \left( \sigma \right)$ is a non-empty, compact, and convex polytope as well. $\square$

In the next example, we use Theorem 7.2 to determine whether a given distribution over posterior belief profiles belongs to $\Sigma (S).$

### Example 7.4

Recall the distribution over posterior belief profiles $\sigma$ in Example 7.1 with

$$\begin{aligned} \text {supp}(\sigma )&= \left\{ \lambda ^1,\lambda ^2,\lambda ^3,\lambda ^4\right\} \\&= \left\{ \left( ({\frac{1}{2}},{\frac{1}{2}}), ({\frac{1}{2}},{\frac{1}{2}}), (0,1)\right) , \left( ({\frac{1}{2}},{\frac{1}{2}}), (0,1), ({\frac{1}{2}},{\frac{1}{2}})\right) , \left( (0,1), ({\frac{1}{2}},{\frac{1}{2}}), ({\frac{1}{2}},{\frac{1}{2}})\right) , \left( (0,1),(0,1),(0,1)\right) \right\} , \end{aligned}$$

and, we have $\sigma (\lambda ^{1}) = \sigma (\lambda ^{2}) = \sigma (\lambda ^{3}) = 1/6$ and $\sigma (\lambda ^{4}) = 1/2.$

Suppose $\sigma \in \Sigma (S).$ Then, by Theorem 7.2 there exists $p \in P(\sigma )$ such that

$$\begin{aligned} p\left( X,\lambda ^1\right) +p\left( X,\lambda ^2\right) =p\left( X,\lambda ^1\right) +p\left( X,\lambda ^3\right) =p\left( X,\lambda ^2\right) +p\left( X,\lambda ^3\right)&=\frac{1}{6}, \\ p\left( X,\lambda ^1\right) +p\left( X,\lambda ^4\right) =p\left( X,\lambda ^2\right) +p\left( X,\lambda ^4\right) =p\left( X,\lambda ^3\right) +p\left( X,\lambda ^4\right)&=0, \end{aligned}$$

where we make use of Condition (ii) for $\omega = X.$ From the first line we obtain $p\left( X,\lambda ^1\right) =p\left( X,\lambda ^2\right) =p\left( X,\lambda ^3\right) =1/12$. Combining this with the second, we find $p\left( X,\lambda ^4\right) =-1/12$. Thus, *p* fails to be non-negative and $\sigma \notin \Sigma (S).$ $\triangle$

Proposition 4.2 gives a necessary and sufficient condition for a finite set $R \subseteq \Delta \left( \Omega \right) ^n$ to be a subset of $\text {supp}(\sigma )$ for some $\sigma \in \Sigma (S)$. We will now provide a necessary and sufficient condition for the opposite inclusion, i.e., we characterize those sets $R \subseteq \Delta \left( \Omega \right) ^n$ such that there is some inducible $\sigma \in \Sigma (S)$ whose support is restricted to *R*. We also characterize those sets *R* such that $R=\text {supp}(\sigma )$ for some $\sigma \in \Sigma (S)$.

### Proposition 7.5

Fix a set of message profiles *S* and let the non-empty and finite $R \subseteq \Delta (\Omega )^n$ be such that, for every $i \in N,$ $|S_{i}| \ge |R_{i}|.$ There exists $\sigma \in \Sigma (S)$ with $\rm{supp}(\sigma )$ $\subseteq R$ if and only if there is $p \in \mathbbm {R}^{\Omega \times R}_{+}$ such that

$$\begin{aligned} \begin{array}{llll} (i) & \sum _{\lambda \in R}p(\omega ,\lambda ) =\lambda ^0(\omega ), & \forall \omega \in \Omega , \\ (ii) & \sum _{\lambda ^{\prime } \in R:\lambda ^{\prime }_i=\lambda _i}p(\omega ,\lambda ^{\prime }) = \lambda _i(\omega )\sum _{\omega ^{\prime }\in \Omega }\sum _{\lambda ^{\prime } \in R:\lambda ^{\prime }_i=\lambda _i}p(\omega ^{\prime },\lambda ^{\prime }), & \forall \omega \in \Omega ,~\forall i \in N,~\forall \lambda _{i} \in R_{i}. \end{array} \end{aligned}$$

If such *p* exists, then the signal $\pi :\Omega \rightarrow \Delta (R)$ defined by

11 $$\begin{aligned} \pi \left( \lambda \vert \omega \right) =\frac{p(\omega ,\lambda )}{\lambda ^0(\omega )}, \quad \forall \omega \in \Omega ,~\forall \lambda \in R, \end{aligned}$$

is an LIS such that $\rm{supp}(\sigma ^{\pi })\subseteq R$. Moreover, if *p* is such that, for all $\lambda \in R$, $\sum _{\omega \in \Omega }p\left( \lambda ,\omega \right) >0$, then $\rm{supp}\left( \sigma ^{\pi }\right) =R$.

### Proof

Assume that there is $p\in \mathbbm {R}^{\Omega \times R}_{+}$ such (*i*) and (*ii*) hold. Let $\pi :\Omega \rightarrow \Delta (R)$ be as defined in (11). We have that

$$\begin{aligned} \sum _{\lambda ^{\prime } \in R} \pi (\lambda ^{\prime } \vert \omega ){\mathop {=}\limits ^{(11)}}\sum _{\lambda ^{\prime } \in R}\frac{p(\omega ,\lambda ^{\prime })}{\lambda ^0(\omega )}{\mathop {=}\limits ^{(i)}}\frac{\lambda ^0(\omega )}{\lambda ^0(\omega )}=1, \quad \forall \omega \in \Omega . \end{aligned}$$

Moreover, for every $\omega \in \Omega ,$ $i \in N,$ and $\lambda _{i} \in S_i^{\pi }$, it holds that

$$\begin{aligned} \frac{\sum _{\lambda ^{\prime } \in R : \lambda ^{\prime }_i = \lambda _i} \pi (\lambda ^{\prime } \vert \omega ) \lambda ^0(\omega )}{\sum _{\omega ^{\prime }\in \Omega }\sum _{\lambda ^{\prime } \in R:\lambda ^{\prime }_i=\lambda _i}\pi (\lambda ^{\prime }\vert \omega ^{\prime }) \lambda ^0(\omega ^{\prime })}&{\mathop {=}\limits ^{(11)}}\frac{\sum _{\lambda ^{\prime } \in R: \lambda ^{\prime }_i=\lambda _i}p(\omega ,\lambda ^{\prime })}{\sum _{\omega ^{\prime } \in \Omega }\sum _{\lambda ^{\prime }\in R:\lambda ^{\prime }_i=\lambda _i}p(\omega ^{\prime },\lambda ^{\prime })} \\&{\mathop {=}\limits ^{(ii)}}\frac{\lambda _i(\omega )\sum _{\omega ^{\prime } \in \Omega } \sum _{\lambda ^{\prime } \in R:\lambda ^{\prime }_i=\lambda _i} p(\omega ^{\prime },\lambda ^{\prime })}{\sum _{\omega ^{\prime } \in \Omega }\sum _{\lambda ^{\prime } \in R : \lambda ^{\prime }_i=\lambda _i}p(\omega ^{\prime },\lambda ^{\prime })} =\lambda _i(\omega ). \end{aligned}$$

Thus, $\pi$ is an LIS and $\text {supp}(\sigma ^{\pi }) = S^{\pi }\subseteq R$.

To account for message sets $S_i$ that do not allow for language-independent messages, note that, for all $i\in N$, $|\text {supp}\left( \sigma ^{\pi }_i\right) |\le |R_i| \le |S_i|$. For every $i \in N$ let $T_{i}$ be a subset of $S_{i}$ with $|T_i|= |\text {supp}\left( \sigma ^{\pi _i}\right) |$ and take a bijection $\psi _{i}: \text {supp}\left( \sigma ^{\pi }_i\right) \rightarrow T_{i}$. Let the signal $\pi ^{\prime }: \Omega \rightarrow \Delta (S)$ be defined by

$$\begin{aligned} \pi ^{\prime }(\psi (\lambda ) | \omega ) = \pi (\lambda | \omega ), \quad \forall \omega \in \Omega ,~\forall \lambda \in \text {supp}\left( \sigma ^{\pi }\right) . \end{aligned}$$

It holds that $\pi \sim \pi ^{\prime },$ so by Proposition 6.7 we have that $\sigma ^{\pi ^{\prime }} = \sigma ^{\pi }$ and $\text {supp}(\sigma ^{\pi ^{\prime }}) = \text {supp}(\sigma ^{\pi })\subseteq R.$

Now assume that $\sigma \in \Sigma (S)$ is such that $\text {supp}(\sigma ) \subseteq R.$ Then, by Theorem 7.2, there is an LIS $\pi : \Omega \rightarrow \Delta (R)$ that induces $\sigma$. Let

12 $$\begin{aligned} p\left( \omega ,\lambda \right) = \pi \left( \lambda |\omega \right) \lambda ^0\left( \omega \right) , \qquad \forall \omega \in \Omega ,~\forall \lambda \in R. \end{aligned}$$

By construction, $S^{\pi }=\text {supp}(\sigma )\subseteq R$ and $p\left( \omega ,\lambda \right) =0$ for all $\lambda \in R{\setminus } S^{\pi }$ and all $\omega \in \Omega$. So, (*i*) is satisfied since

$$\begin{aligned} \sum _{\lambda ^{\prime } \in R} p(\omega , \lambda ^{\prime }) {\mathop {=}\limits ^{(12)}}\sum _{\lambda ^{\prime } \in R} \pi (\lambda ^{\prime } \vert \omega ) \lambda ^0(\omega ) = \lambda ^0(\omega ) \sum _{\lambda ^{\prime } \in S^{\pi }} \pi (\lambda ^{\prime } \vert \omega ) = \lambda ^0(\omega ), \quad \forall \omega \in \Omega . \end{aligned}$$

Further, for every $\omega \in \Omega ,$ $i \in N,$ and $\lambda _{i} \in R_{i},$ it holds that

$$\begin{aligned} \sum _{\lambda ^{\prime } \in R : \lambda ^{\prime }_i=\lambda _i} p(\omega ,\lambda ^{\prime })&{\mathop {=}\limits ^{(12)}}\sum _{\lambda ^{\prime } \in R : \lambda ^{\prime }_i = \lambda _i} \pi (\lambda ^{\prime } \vert \omega ) \lambda ^0(\omega ) =\pi _i\left( \lambda _i|\omega \right) \lambda ^0\left( \omega \right) \\&{\mathop {=}\limits ^{(1)}}\lambda _i(\omega ) \sum _{\omega ^{\prime } \in \Omega } \pi _i(\lambda _i \vert \omega ^{\prime }) \lambda ^0(\omega ^{\prime }) = \lambda _i(\omega ) \sum _{\omega ^{\prime } \in \Omega } \sum _{\lambda ^{\prime } \in R : \lambda ^{\prime }_i = \lambda _i} \pi (\lambda ^{\prime } \vert \omega ^{\prime }) \lambda ^0(\omega ^{\prime }) \\&{\mathop {=}\limits ^{(12)}}\lambda _i(\omega ) \sum _{\omega ^{\prime } \in \Omega } \sum _{\lambda ^{\prime } \in R : \lambda ^{\prime }_i = \lambda _i} p(\omega ^{\prime }, \lambda ^{\prime }). \end{aligned}$$

Hence, (*ii*) is satisfied.

Lastly, let *p* be such that, for all $\lambda \in R$, $\sum _{\omega \in \Omega }p\left( \lambda ,\omega \right) >0$. Then for each $\lambda \in R$, there is $\omega \in \Omega$ such that $\pi \left( \lambda |\omega \right) >0$. Thus, $\text {supp}\left( \sigma ^{\pi }\right) =S^{\pi }=R$. $\square$

As $\pi$ is defined by (11), (*i*) ensures that $\pi \left( \cdot |\omega \right) \in \Delta (\Omega )^{n}$ for all $\omega \in \Omega$ so that $\pi$ is a signal. Condition (*ii*) ensures correct belief updating: as before, the left-hand side is the probability that *i* has belief $\lambda _i$ and the true state is $\omega$; the right-hand side is the product of the probability that the state is $\omega$ conditional on *i*’s having belief $\lambda _i$ and the probability that *i* has belief $\lambda _i$.

In our discussion of Proposition 6.7, stating that equivalent signals induce the same distribution, we announced that the converse need not be true. We can now easily provide the required counterexample.

### Example 7.6

Let $N = \{1,2\},$ $\Omega = \{X,Y\},$ $\lambda ^{0}(X) = 1/3,$ and $S = \Delta (\Omega )^{n}.$ Consider the distribution $\sigma$ defined by

$$\begin{aligned} \text {supp}(\sigma ^{\pi })&=\left\{ \lambda ^{1},\lambda ^{2},\lambda ^{3},\lambda ^{4}\right\} \\&=\left\{ (({\frac{1}{2}},{\frac{1}{2}}), ({\frac{1}{2}},{\frac{1}{2}})), (({\frac{1}{2}},{\frac{1}{2}}),({\frac{1}{4}},{\frac{3}{4}})), (({\frac{1}{4}},{\frac{3}{4}}),({\frac{1}{2}},{\frac{1}{2}})), (({\frac{1}{4}},{\frac{3}{4}}),({\frac{1}{4}},{\frac{3}{4}}))\right\} , \end{aligned}$$

$\sigma (\lambda ^{1}) = \sigma (\lambda ^{2}) = \sigma (\lambda ^{3}) = 1/6$ and $\sigma (\lambda ^{4}) = 1/2.$ One can easily verify that $p, p^{\prime } \in \mathbb {R}^{\Omega \times \text {supp}(\sigma )}_{+}$ defined by

**Table:** 

$p(\omega ,\lambda )$	$\lambda ^1$	$\lambda ^2$	$\lambda ^3$	$\lambda ^4$
*X*	$\frac{1}{12}$	$\frac{1}{12}$	$\frac{1}{12}$	$\frac{1}{12}$
*Y*	$\frac{1}{12}$	$\frac{1}{12}$	$\frac{1}{12}$	$\frac{5}{12}$

**Table:** 

$p'(\omega ,\lambda )$	$\lambda ^1$	$\lambda ^2$	$\lambda ^3$	$\lambda ^4$
*X*	$\frac{1}{6}$	0	0	$\frac{1}{6}$
*Y*	0	$\frac{1}{6}$	$\frac{1}{6}$	$\frac{1}{3}$

are both solutions to the system of equations in Theorem 7.2. We define $\pi , \pi ^{\prime } \in \Pi ^{\ell }(S)$ by applying (8) to *p* and $p^{\prime },$ respectively, that is,

**Table:** 

$\pi (\lambda | \omega )$	$\lambda ^1$	$\lambda ^2$	$\lambda ^3$	$\lambda ^4$
*X*	$\frac{1}{4}$	$\frac{1}{4}$	$\frac{1}{4}$	$\frac{1}{4}$
*Y*	$\frac{1}{8}$	$\frac{1}{8}$	$\frac{1}{8}$	$\frac{5}{8}$

**Table:** 

$\pi '(\lambda | \omega )$	$\lambda ^{1}$	$\lambda ^{2}$	$\lambda ^{3}$	$\lambda ^{4}$
*X*	$\frac{1}{2}$	0	0	$\frac{1}{2}$
*Y*	0	$\frac{1}{4}$	$\frac{1}{4}$	$\frac{1}{2}$

Both $\pi$ and $\pi '$ induce $\sigma .$ Yet, as $\pi \ne \pi ^{\prime },$ Proposition 6.8 implies that $\pi$ and $\pi ^{\prime }$ are not equivalent. $\triangle$

## The information and posterior correspondences

Our objective in this section is to provide a framework in which we can analyze what receivers know about each other’s messages, so that we can later answer the question of how a sender can make sure that receivers know “as little as possible”. We follow the standard approach based on information correspondences, as in, for instance, Osborne and Rubinstein (1994), Chapter 5.

Given a signal $\pi \in \Pi (S),$ we refer to an element $(\omega ,s) \in \Omega \times S^{\pi }$ such that $\pi (s\vert \omega ) > 0$ as a *history* and to an element $(\omega ,\lambda ) \in \Omega \times \text {supp}(\sigma ^{\pi })$ such that there exists $s \in S^{\pi }$ with $\pi (s \vert \omega ) > 0$ and $\lambda ^{\pi ,s} = \lambda$ as a *posterior history*. We denote the sets of histories and posterior histories, respectively, by

$$\begin{aligned} H^{\pi }&=\left\{ (\omega ,s)\in \Omega \times S^{\pi }\vert \,\pi (s\vert \omega )>0\right\} ,\\ \Lambda ^{\pi }&=\left\{ \left( \omega ,\lambda \right) \in \Omega \times \Delta (\Omega )^n \vert \exists s \in S^{\pi } \text{ such } \text{ that } \pi (s\vert \omega )>0 \text{ and } \lambda ^{\pi ,s} = \lambda \right\} \\&=\left\{ \left( \omega ,\lambda ^{\pi ,s}\right) \in \Omega \times \Delta (\Omega )^n\vert (\omega ,s)\in H^\pi \right\} . \end{aligned}$$

Note that if $\pi \in \Pi ^{\ell }(S),$ then $H^{\pi } = \Lambda ^{\pi }.$

### Example 8.1

Recall $\pi$ and $\pi '$ from Example 7.6. The sets of possible histories are:

$$\begin{aligned} H^{\pi }&= \left\{ \left( X,\lambda ^1\right) , \left( X,\lambda ^2\right) , \left( X,\lambda ^3\right) , \left( X,\lambda ^4\right) , \left( Y,\lambda ^1\right) , \left( Y,\lambda ^2\right) , \left( Y,\lambda ^3\right) , \left( Y,\lambda ^4\right) \right\} \\ H^{\pi '}&=\left\{ \left( X,\lambda ^1\right) , \left( X,\lambda ^4\right) , \left( Y,\lambda ^2\right) , \left( Y,\lambda ^3\right) , \left( Y,\lambda ^4\right) \right\} . \end{aligned}$$

As both signals are language-independent, we have $\Lambda ^{\pi }=H^{\pi }$ and $\Lambda ^{\pi '}=H^{\pi '}$. $\triangle$

We next introduce the standard notion of an information correspondence.

### Definition 8.2

Fix a set of message profiles *S* and let $\pi \in \Pi (S).$ The *information correspondence* $P^{\pi }_i: H^{\pi }\rightrightarrows H^{\pi }$ of $i \in N$ is defined as

$$\begin{aligned} P^{\pi }_i(\omega ,s)=\left\{ (\omega ',s')\in H^{\pi }\vert \, s'_i=s_i\right\} , \quad \forall (\omega ,s) \in H^{\pi }. \end{aligned}$$

That is, $P^{\pi }_i(\omega ,s)$ is the set of histories receiver *i* considers possible when the true history is $(\omega ,s)$. As we call $P^{\pi }_i$ an information correspondence, it seems appropriate to briefly show that this name is deserved, i.e., consistent with the common definition of an information correspondence.

### Lemma 8.3

Fix a set of message profiles *S* and let $\pi \in \Pi (S)$ and $i \in N.$ The information correspondence $P^{\pi }_i$ satisfies the following two conditions:

1. For all $$(\omega ,s)\in H^{\pi }$$, $$(\omega ,s)\in P^{\pi }_i(\omega ,s)$$.
2. If $$(\omega ',s')\in P^{\pi }_i(\omega ,s)$$, then $$P^{\pi }_i(\omega ',s')=P^{\pi }_i(\omega ,s)$$.

### Proof

Let $(\omega ,s) \in H^{\pi }.$ Suppose $(\omega ,s)\notin P^{\pi }_i(\omega ,s)$. Then, $s_i\ne s_i$, a contradiction. Thus, C1 is satisfied.

Next, let $(\omega ',s')\in P^{\pi }_i(\omega ,s)$ and $(\omega '',s'')\in P^{\pi }_i(\omega ',s')$. Then, $s''_i=s'_i= s_i$, so $(\omega '',s'')\in P^{\pi }_i(\omega ,s)$, and consequently, $P^{\pi }_i(\omega ',s')\subseteq P^{\pi }_i(\omega ,s)$. Since $s_i'=s_i$, it holds that $(\omega ,s)\in P^{\pi }_i(\omega ',s')$ as well, and the same arguments imply $P^{\pi }_i(\omega ,s)\subseteq P^{\pi }_i(\omega ',s')$. So, C2 is satisfied. $\square$

It should be noted that an information correspondence satisfies conditions C1 and C2 if and only if it partitions the set of histories into information sets (see Osborne and Rubinstein, 1994, Lemma 68.3). In our case, we can use $P_i^{\pi }$ to define a partition of the set $H^{\pi }$ as

$$\begin{aligned} \mathcal P^{\pi }_i=\left\{ P^{\pi }_i(\omega ,s)\vert \,(\omega ,s)\in H^{\pi }\right\} . \end{aligned}$$

This partition reflects *i*’s knowledge about the true history: whenever the true history is $\left( \omega ,s\right)$, *i* knows that the true history lies in $P^{\pi }_i(\omega ,s)$.

### Example 8.4

Recall $\pi$ in Example 5.2. The information correspondence partitions the set of histories as follows:

$$\begin{aligned} P^{\pi }_1(X,(v,x))=P^{\pi }_1(Y,(v,y))&=\left\{ (X,(v,x)),(Y,(v,y))\right\} ,\\ P^{\pi }_1(X,(w,w))=P^{\pi }_1(Y,(w,w))&=\left\{ (X,(w,w)),(Y,(w,w))\right\} ,\\ \\ P^{\pi }_2(X,(v,x))&=\left\{ (X,(v,x)\right\} ,\nonumber \\ P^{\pi }_2(Y,(v,y))&=\left\{ (Y,(v,y))\right\} ,\nonumber \\ P^{\pi }_2(X,(w,w))=P^{\pi }_2(Y,(w,w))&=\left\{ (X,(w,w)),(Y,(w,w))\right\} . \end{aligned}$$

Now consider $\pi ^{\prime }$ in Example 5.4. The information correspondence partitions the set of histories as follows:

$$\begin{aligned} P^{\pi ^{\prime }}_1(X,(w,x))&=P^{\pi ^{\prime }}_1(Y,(w,y))=P^{\pi ^{\prime }}_1(X,(w,w))=P^{\pi ^{\prime }}_1(Y,(w,w))\nonumber \\&=\left\{ (X,(w,x)),(Y,(w,y)),(X,(w,w)),(Y,(w,w))\right\} ,\\ \\ P^{\pi ^{\prime }}_2(X,(w,x))&=\left\{ (X,(w,x)\right\} ,\nonumber \\ P^{\pi ^{\prime }}_2(Y,(w,y))&=\left\{ (Y,(w,y))\right\} ,\nonumber \\ P^{\pi ^{\prime }}_2(X,(w,w))=P^{\pi ^{\prime }}_2(Y,(w,w))&=\left\{ (X,(w,w)),(Y,(w,w))\right\} . \end{aligned}$$

It is easy to verify that both C1 and C2 are satisfied. In particular, the information partitions of $\mathcal P_i^\pi$ and, respectively, $\mathcal P_i^{\pi '}$ are given by

$$\begin{aligned} \mathcal P^{\pi }_1&=\left\{ \left\{ (X,(v,x)),(Y,(v,y))\right\} ,\left\{ (X,(w,w)),(Y,(w,w))\right\} \right\} , \\ \mathcal P^{\pi }_2&=\left\{ \left\{ (X,(v,x))\right\} ,\left\{ (Y,(v,y))\right\} ,\left\{ (X,(w,w)),(Y,(w,w))\right\} \right\} , \\ \\ \mathcal P^{\pi ^{\prime }}_1&=\left\{ \left\{ (X,(w,x)),(Y,(w,y)),(X,(w,w)),(Y,(w,w))\right\} \right\} , \\ \mathcal P^{\pi ^{\prime }}_2&=\left\{ \left\{ (X,(w,x))\right\} ,\left\{ (Y,(w,y))\right\} ,\left\{ (X,(w,w)),(Y,(w,w))\right\} \right\} . \end{aligned}$$

$$\triangle$$

Even though $\pi$ and $\pi '$ in Example 8.4 induce the same distribution, it is not possible to compare their information partitions since they employ different messages and thus have distinct sets of histories. Still, we can compare such signals via the sets of possible posterior histories of receivers.

### Definition 8.5

Fix a set of message profiles *S* and let $\pi \in \Pi (S).$ The *posterior correspondence* $Q^{\pi }_i: H^{\pi }\rightrightarrows \Lambda ^{\pi }$ of $i \in N$ is defined as

$$\begin{aligned} Q^{\pi }_i(\omega ,s)&= \{(\omega ',\lambda ^{s'})\in \Lambda ^{\pi }|\,(\omega ',s')\in P_i^{\pi }(\omega ,s)\}, \quad \forall (\omega ,s) \in H^{\pi }. \end{aligned}$$

The set $Q^{\pi }_i(\omega ,s)$ contains all posterior histories *i* deems possible if the true history is $(\omega ,s)$.^16^

### Example 8.6

Recall the information correspondences in Example 8.4. The posterior correspondences related to $\pi$ are as follows.

$$\begin{aligned} Q^{\pi }_1(X,(v,x))=Q^{\pi }_1(Y,(v,y))&=\left\{ \left( X,\left( {\frac{1}{2}},1\right) \right) ,\left( Y,\left( {\frac{1}{2}},0\right) \right) \right\} , \\ Q^{\pi }_1(X,(w,w))=Q^{\pi }_1(Y,(w,w))&=\left\{ \left( X,\left( {\frac{1}{2}},{\frac{1}{2}}\right) \right) ,\left( Y,\left( {\frac{1}{2}},{\frac{1}{2}}\right) \right) \right\} , \\ \\ Q^{\pi }_2(X,(v,x))&=\left\{ \left( X,\left( {\frac{1}{2}},1\right) \right) \right\} , \\ Q^{\pi }_2(Y,(v,y))&=\left\{ \left( Y,\left( {\frac{1}{2}},0\right) \right) \right\} , \\ Q^{\pi }_2(X,(w,w))=Q^{\pi }_2(Y,(w,w))&=\left\{ \left( X,\left( {\frac{1}{2}},{\frac{1}{2}}\right) \right) ,\left( Y,\left( {\frac{1}{2}},{\frac{1}{2}}\right) \right) \right\} . \end{aligned}$$

The posterior correspondences related to $\pi ^{\prime }$ are as follows.

$$\begin{aligned} Q^{\pi ^{\prime }}_1(X,(w,x))&=Q^{\pi ^{\prime }}_1(Y,(w,y))=Q^{\pi ^{\prime }}_1(X,(w,w))=Q^{\pi ^{\prime }}_1(Y,(w,w))\nonumber \\& \quad =\left\{ \left( X,\left( {\frac{1}{2}},1\right) \right) ,\left( Y,\left( {\frac{1}{2}},0\right) \right) ,\left( X,\left( {\frac{1}{2}},{\frac{1}{2}}\right) \right) ,\left( Y,\left( {\frac{1}{2}},{\frac{1}{2}}\right) \right) \right\} , \\ \\ Q^{\pi ^{\prime }}_2(X,(w,x))&=\left\{ \left( X,\left( {\frac{1}{2}},1\right) \right) \right\} , \\ Q^{\pi ^{\prime }}_2(Y,(w,y))&=\left\{ \left( Y,\left( {\frac{1}{2}},0\right) \right) \right\} , \\ Q^{\pi ^{\prime }}_2(X,(w,w))=Q^{\pi ^{\prime }}_2(Y,(w,w))&=\left\{ \left( X,\left( {\frac{1}{2}},{\frac{1}{2}}\right) \right) ,\left( Y,\left( {\frac{1}{2}},{\frac{1}{2}}\right) \right) \right\} . \end{aligned}$$

One can easily see that there is a bijection between the set of histories and the set of posterior histories for both $\pi$ and $\pi '$ that preserves the partition structure. $\triangle$

For $\pi \in \Pi (S)$ and $i\in N,$ define $\mathcal Q^{\pi }_i=\left\{ Q^{\pi }_i(\omega ,s)\vert \,(\omega ,s)\in H^{\pi }\right\}$. Note that in Example 8.6 both $\mathcal Q^{\pi }_i$ and $\mathcal Q^{\pi '}_i$ are partitions for any $i \in N.$ However, this is not always true.

### Example 8.7

Let $N=\left\{ 1,2\right\}$, $\Omega =\left\{ X,Y\right\}$, and $\lambda ^0(X)=1/3$. Let signal $\pi \in \Pi (S)$ be given as follows:

**Table:** 

$\pi$	(*x*, *x*)	(*x*, *y*)	(*y*, *x*)	(*y*, *y*)	(*a*, *a*)	(*a*, *b*)	(*b*, *a*)	(*b*, *b*)
*X*	$\frac{1}{6}$	0	0	$\frac{1}{6}$	$\frac{1}{6}$	$\frac{1}{6}$	$\frac{1}{6}$	$\frac{1}{6}$
*Y*	0	$\frac{1}{12}$	$\frac{1}{12}$	$\frac{1}{6}$	$\frac{1}{12}$	$\frac{1}{12}$	$\frac{1}{12}$	$\frac{5}{12}$

For the posterior correspondence we find

$$\begin{aligned}&Q^{\pi }_1(X,(x,x))=\left\{ \left( X,\left( {\frac{1}{2}},{\frac{1}{2}}\right) \right) ,\left( Y,\left( {\frac{1}{2}},{\frac{1}{4}}\right) \right) \right\} , \\&Q^{\pi }_1(X,(a,a))=\left\{ \left( X,\left( {\frac{1}{2}},{\frac{1}{2}}\right) \right) ,\left( X,\left( {\frac{1}{2}},{\frac{1}{4}}\right) \right) ,\left( Y,\left( {\frac{1}{2}},{\frac{1}{2}}\right) \right) ,\left( Y,\left( {\frac{1}{2}},{\frac{1}{4}}\right) \right) \right\} . \end{aligned}$$

Since $Q^{\pi }_1(X,(x,x))\ne Q^{\pi }_1(X,(a,a))$ and $\left( X,\left( 1/2,1/2\right) \right) \in Q^{\pi }_1(X,(x,x))\cap Q^{\pi }_1(X,(a,a))$, $\mathcal Q^{\pi }_1$ is not a partition. $\triangle$

The reason why $Q^{\pi }_1$ in Example 8.7 is not a partition is that message profiles (*x*, *x*) and (*a*, *a*) lead to the same posterior belief profile, yet $\left( x,x\right)$ realizes only in state *X* whereas $\left( a,a\right)$ realizes in both states. This situation, of course, can happen only as long as the signal is not minimal. Thus, $\pi \in \Pi^{\mathrm{m}}(S)$ is sufficient for $\mathcal Q^{\pi }_i$ to be a partition for all $i\in N$. To prove this we define, for $\pi \in \Pi (S),$ the (surjective) map $\phi : H^{\pi } \rightarrow \Lambda ^{\pi }$ by

13 $$\begin{aligned} \phi (\omega ,s) = (\omega , \lambda ^{\pi ,s}), \quad \forall (\omega ,s) \in H^{\pi }. \end{aligned}$$

### **Proposition 8.8**

Fix a set of message profiles *S* and let $\pi \in \Pi ^\mathrm{{m}}(S)$. Then $\phi$ is a bijection and, for every $(\omega ,s),(\omega ',s')\in H^{\pi }$ and every $i\in N$, it holds that $(\omega ,s)\in P^{\pi }_i(\omega ',s')$ if and only if $\phi (\omega ,s)\in Q^{\pi }_i(\omega ',s')$. In particular, $\mathcal Q^{\pi }_i$ is a partition.

### Proof

First note that since $\pi \in \Pi ^\mathrm{{m}}(S)$, for any $(\omega ,s),(\omega ',s')\in H^{\pi }$ with $s\ne s'$, it holds that $\left( \omega ,\lambda ^{\pi ,s}\right) \ne \left( \omega ',\lambda ^{\pi ,s'}\right)$. That is, no two distinct histories are mapped to the same posterior history. Thus, $\phi$ is a bijection.

Let $(\omega ,s),(\omega ',s')\in H^{\pi }$ and $i \in N.$ If $(\omega ,s)\in P^{\pi }_i(\omega ',s')$, then $\phi \left( \omega ,s\right) =\left( \omega ,\lambda ^{\pi ,s}\right) \in Q^{\pi }_i\left( \omega ',s'\right)$ by the definition of $Q^{\pi }_i\left( \omega ',s'\right)$. If $\left( \omega ,\lambda ^{\pi ,s}\right) =\phi (\omega ,s)\in Q^{\pi }_i(\omega ',s')$, then $(\omega ,s)\in P^{\pi }_i(\omega ',s')$. Therefore, $(\omega ,s)\in P^{\pi }_i(\omega ',s')$ if and only if $\phi (\omega ,s)\in Q^{\pi }_i(\omega ',s')$.

Suppose $Q_i^{\pi }\left( \omega ,s\right) \cap Q_i^{\pi }\left( \omega ',s'\right) \ne \emptyset$. It follows that $P_i^{\pi }\left( \omega ,s\right) \cap P_i^{\pi }\left( \omega ',s'\right) \ne \emptyset$, so $P_i^{\pi }\left( \omega ,s\right) = P_i^{\pi }\left( \omega ',s'\right)$. Therefore, $Q_i^{\pi }\left( \omega ,s\right) =\phi \left( P_i^{\pi }\left( \omega ,s\right) \right) = \phi \left( P_i^{\pi }\left( \omega ',s'\right) \right) = Q_i^{\pi }\left( \omega ',s'\right) ,$ so $\mathcal Q^{\pi }_i$ is a partition. $\square$

The converse of Proposition 8.8 is not true. That is, even if the map $\phi$ in (13) is a bijection with the required properties, it is still possible that $\pi$ is not minimal.

### Example 8.9

Let $N=\left\{ 1,2\right\}$, $\Omega =\left\{ X,Y\right\}$, and $\lambda ^0(X)=1/3$. Let the signal $\pi \in \Pi (S)$ be defined by

**Table:** 

$\pi$	(*a*, *a*)	(*b*, *b*)	(*a*, *c*)	(*c*, *a*)	(*b*, *d*)	(*d*, *b*)	(*e*, *e*)
*X*	$\frac{1}{6}$	0	0	0	$\frac{1}{4}$	$\frac{1}{6}$	$\frac{5}{12}$
*Y*	0	$\frac{1}{4}$	$\frac{1}{6}$	$\frac{1}{4}$	0	0	$\frac{1}{3}$

Then, for receiver 1 we have $\lambda ^{\pi ,(a,a)}_1(X)=\lambda ^{\pi ,(b,b)}_1(X)=1/3$, $\lambda ^{\pi ,(c,a)}_1(X)=0$, $\lambda ^{\pi ,(d,b)}_1(X)=1$, and $\lambda ^{\pi ,(e,e)}_1(X)=5/13$. For receiver 2 we have $\lambda ^{\pi ,(a,a)}_2(X)=\lambda ^{\pi ,(b,b)}_2(X)=1/4$, $\lambda ^{\pi ,(a,c)}_2(X)=0$, $\lambda ^{\pi ,(b,d)}_2(X)=1$, and $\lambda ^{\pi ,(e,e)}_2(X)=5/13$. Note that message profiles (*a*, *a*) and (*b*, *b*) lead to the same posterior belief profile, (1/3, 1/4). Thus, $\pi$ is not minimal. For the support of the induced distribution $\sigma$, we find

$$\begin{aligned} \text {supp}(\sigma )=\left\{ \left( {\frac{1}{3}},{\frac{1}{4}}\right) ,\left( {\frac{1}{3}},0\right) ,\left( 0,{\frac{1}{4}}\right) ,\left( {\frac{1}{3}},1\right) ,\left( 1,{\frac{1}{4}}\right) ,\left( {\frac{5}{13}},{\frac{5}{13}}\right) \right\} . \end{aligned}$$

The sets $\mathcal{P}^{\pi }_{1}$ and $\mathcal{Q}^{\pi }_{1}$ defined by the information and posterior correspondences of receiver 1 are as follows:

$$\begin{aligned} \mathcal P^{\pi }_1=&\left\{ \left\{ (X,(a,a)),(Y,(a,c))\right\} ,\left\{ (Y,(c,a))\right\} ,\left\{ (X,(b,d)),(Y,(b,b))\right\} ,\left\{ (X,(d,b))\right\} ,\right. \\&\left. \left\{ (X,(e,e)),(Y,(e,e))\right\} \right\} , \\ \mathcal Q^{\pi }_1=&\left\{ \left\{ \left( X,\left( {\frac{1}{3}},{\frac{1}{4}}\right) \right) ,\left( Y,\left( {\frac{1}{3}},0\right) \right) \right\} ,\left\{ \left( Y,\left( 0,{\frac{1}{4}}\right) \right) \right\} ,\left\{ \left( X,\left( {\frac{1}{3}},1\right) \right) ,\left( Y,\left( {\frac{1}{3}},{\frac{1}{4}}\right) \right) \right\} ,\left\{ \left( X,\left( 1,{\frac{1}{4}}\right) \right) \right\} ,\right. \\&\left. \left\{ \left( X,\left( {\frac{5}{13}},{\frac{5}{13}}\right) \right) ,\left( Y,\left( {\frac{5}{13}},{\frac{5}{13}}\right) \right) \right\} \right\} . \end{aligned}$$

Similar calculations can be made for receiver 2. It is easily checked that not only are $\mathcal Q_1^{\pi }$ and $\mathcal{Q}^{\pi }_{2}$ partitions, but $\phi$ is a bijection as well. The reason $\mathcal Q^{\pi }_1$ and $\mathcal{Q}^{\pi }_{2}$ are partitions, even though $\pi \notin \Pi ^\mathrm{{m}}(S),$ is that the message profiles which lead to the same posterior, (*a*, *a*) and (*b*, *b*), never realize in the same state. $\triangle$

Observe that if $\pi \in \Pi ^\ell (S),$ then $\phi$ in (13) is the identity. Hence, Proposition 8.8 implies that the partitions $\mathcal P_i^{\pi }$ and $\mathcal Q_i^{\pi }$ are identical. For all $\pi \in \Pi ^\mathrm{{i}}(S)$, let $\pi ^{\ell }\in \Pi ^{\ell }(S)$ be defined as in (7), i.e., $\pi ^{\ell }$ denotes the LIS obtained by replacing the messages of $\pi$ by the posteriors they lead to. Then the posterior history partition of $\pi$ is equal to the history partition of $\pi ^{\ell }$. Thus, we have the following corollary.

### Corollary 8.10

Fix a set of message profiles *S* and let $\pi \in \Pi ^\mathrm{{i}}(S)$ and $\pi ^{\ell } \in \Pi ^{\ell }(S)$ be defined as in (7). Then, for all $i\in N$, $\mathcal Q^{\pi }_i=\mathcal Q^{\pi ^{\ell }}_i=\mathcal P^{\pi ^{\ell }}_i$.

## Informativeness of signals

Example 8.6 derives the posterior correspondences of the receivers under $\pi$ and $\pi '$ from Examples 5.2 and 5.4. Observe that receiver 1 has more precise information about receiver 2’s knowledge of the true state under $\pi$: while he only observes *w* under $\pi ^{\prime }$ and, thus, never learns what message receiver 2 has observed, under $\pi$ upon observing *v* he knows that receiver 2 knows the true state. In this sense $\pi$ is “more informative”: a notion that depends on the posterior correspondence and which we will make more formal soon. Beforehand, we make the brief observation that the posterior correspondence itself is invariant under equivalence. This stems from the fact that equivalence does not only require identical posteriors, but also identical information sets. In particular, under two equivalent signals a receiver will have identical higher order knowledge about everybody’s beliefs of any order.

### Lemma 9.1

Fix a set of message profiles *S* and let $\pi ,\pi '\in \Pi (S)$ with $\pi \sim \pi '$. Then, for every $i \in N,$ $\mathcal Q_i^{\pi }=\mathcal Q_i^{\pi '}.$

### Proof

Since $\pi \sim \pi '$, for every $i \in N$ there is a bijection $\psi _{i}: S^{\pi }_{i} \rightarrow S^{\pi ^{\prime }}_{i}$ such that, for every $\omega \in \Omega ,$ for every $s \in S^{\pi },$ $\pi ^{\prime }(\psi (s) | \omega ) = \pi (s | \omega ).$

Let $$(\omega ,s) \in H^{\pi }$$ and $$i \in N.$$

We have that $(\omega ^{\prime }, s^{\prime }) \in P^{\pi }_{i}(\omega ,s)$ if and only if $(\omega ^{\prime },s^{\prime }) \in H^{\pi }$ and $s^{\prime }_{i} = s_{i}$, which is equivalent to $(\omega ^{\prime }, \psi (s^{\prime })) \in H^{\pi ^{\prime }}$ and $\psi _{i}(s^{\prime }_{i}) = \psi _{i}(s_{i})$, which in turn is equivalent to $(\omega ^{\prime }, \psi (s^{\prime })) \in P^{\pi ^{\prime }}_{i}(\omega , \psi (s)).$

Let $\left( \omega ',\lambda '\right) \in Q_i^{\pi '}\left( \omega ,\psi \left( s\right) \right)$. Then, by the definition of $Q_i^{\pi '}$, there is $\left( \omega ',\psi \left( s'\right) \right) \in P_i^{\pi '}\left( \omega ,\psi \left( s\right) \right)$ with $\lambda ^{\pi ',\psi \left( s^{\prime }\right) } = \lambda ^{\prime }.$ As shown in the previous paragraph, this implies $\left( \omega ',s'\right) \in P_i^{\pi }\left( \omega ,s\right)$. Since by construction $\lambda ^{\pi ,s'}=\lambda ^{\pi ',\psi \left( s'\right) }=\lambda '$, it follows that $\left( \omega ',\lambda '\right) \in Q^{\pi }\left( \omega ,s\right)$ and therefore $Q_i^{\pi '}\left( \omega ,\psi \left( s\right) \right) \subseteq Q^{\pi }_{i}\left( \omega ,s\right)$.

Since $\sim$ is symmetric, we also have that $Q^{\pi }_{i}\left( \omega ,s\right) \subseteq Q_i^{\pi '}\left( \omega ,\psi \left( s\right) \right) .$ $\square$

We argued in the beginning of this section that the signal $\pi$ is “more informative” for receiver 1 than signal $\pi ^{\prime }$; under $\pi$, receiver 1 knows (with positive probability) that receiver 2 knows the true history. In particular, partition $\mathcal Q^\pi _1$ is *finer* than partition $\mathcal Q^{\pi '}_1$, so that receiver 1 can distinguish more between posterior histories. In other words, for any element of $Q^\pi _1$ we can find an element of $Q^{\pi '}_1$ that includes the former. We now formalize the definition of being more informative.

### Definition 9.2

Fix a set of message profiles *S*. Let $\sigma \in \Sigma (S)$ and $\pi , \pi ' \in \Pi (\sigma ).$ The signal $\pi '$* is at least as informative as *$\pi$ if for all $i\in N$ it holds that

(i) for all $Q' \in \mathcal Q^{\pi '}_i$ there exists $Q \in \mathcal Q^{\pi }_i$ such that $Q' \subseteq Q$,
(ii) for all $Q \in \mathcal Q^{\pi }_i,Q' \in \mathcal Q^{\pi '}_i$ with $Q \cap Q' \ne \emptyset$ it holds that $Q' \subseteq Q$.

Moreover, $\pi$ and $\pi '$ are *equally informative* if $\pi$ is at least as informative as $\pi '$ and vice versa; $\pi '$ is *more informative than *$\pi$ if $\pi '$ is at least as informative as $\pi$ and not equally informative.

Observe that to compare $\pi$ and $\pi '$ in terms of their informativeness, Definition 9.2 does not require $\mathcal Q^\pi _i$ and $\mathcal Q^{\pi '}_i$ to be partitions: condition (ii) ensures that we are able to compare them even if they are not. When they are partitions, which is the case if $\pi ,\pi '\in \Pi ^\mathrm{{m}}(S)$ by Proposition 8.8, then condition (ii) in Definition 9.2 is redundant.

It is easily verified that the notion of being at least as informative is transitive. Our next observation serves as a sanity check: two signals should be equally informative if and only if they induce the same posterior history. And this is true.

### Lemma 9.3

Fix a set of message profiles *S*. Let $\sigma \in \Sigma (S)$ and $\pi , \pi ' \in \Pi (\sigma ).$ Then $\pi$ and $\pi '$ are *equally informative* if and only if, for every $i \in N,$ $\mathcal{Q}^{\pi }_{i} = \mathcal{Q}^{\pi ^{\prime }}_{i}$.

### Proof

Clearly, if, for every $i \in N,$ $\mathcal Q_i^{\pi }= \mathcal Q_i^{\pi '},$ then $\pi$ and $\pi '$ are equally informative. For the other direction, assume that $\pi$ and $\pi '$ are equally informative. Let $i \in N.$ As $\pi '$ is as informative as $\pi$, for all $Q'\in \mathcal Q_i^{\pi '}$ there is $Q\in \mathcal Q_i^{\pi }$ such that $Q'\subseteq Q$. As $Q'\cap Q\ne \emptyset$ and as $\pi$ is as informative as $\pi '$, it must hold that $Q\subseteq Q'$, i.e., $Q'=Q$. Thus, $\mathcal Q_i^{\pi '}\subseteq \mathcal Q_i^{\pi }$. Using the same arguments one finds $\mathcal Q_i^{\pi }\subseteq \mathcal Q_i^{\pi '}$. $\square$

An immediate consequence of Lemmas 9.1 and 9.3 is that equivalent signals are equally informative. This is in line with our interpretation of equivalent signals as using different languages: if the same messages were conveyed in different languages, one would not expect them to become more or less informative.

In Example 8.6, we found the posterior correspondences under signals $\pi$ and $\pi '$ but did not consider their informativeness. In the next example, we show that $\pi$ is more informative than $\pi '$.

### Example 9.4

Recall the signals $\pi$ and $\pi '$ from Examples 5.2 and 5.4. The posterior history correspondences of $\pi$ and $\pi '$ were derived in Example 8.6. Note that $\Lambda ^\pi =\Lambda ^{\pi '}$ and that $\pi ,\pi '\in \Pi ^\mathrm{{m}}(S)$. Thus, Proposition 8.8 implies that, for every $i\in N$, $\mathcal Q^{\pi }_i$ and $\mathcal Q^{\pi '}_i$ are partitions of the same set. More precisely, they are given as

$$\begin{aligned}&\mathcal Q^{\pi }_1 = \left\{ \left\{ \left( X,\left( {\frac{1}{2}},1\right) \right) , \left( Y,\left( {\frac{1}{2}},0\right) \right) \right\} , \left\{ \left( X,\left( {\frac{1}{2}},{\frac{1}{2}}\right) \right) ,\left( Y,\left( {\frac{1}{2}},{\frac{1}{2}}\right) \right) \right\} \right\} , \\&\mathcal Q^{\pi }_2 = \left\{ \left\{ \left( X,\left( {\frac{1}{2}},1\right) \right) \right\} , \left\{ \left( Y,\left( {\frac{1}{2}},0\right) \right) \right\} , \left\{ \left( X,\left( {\frac{1}{2}},{\frac{1}{2}}\right) \right) , \left( Y,\left( {\frac{1}{2}},{\frac{1}{2}}\right) \right) \right\} \right\} , \\ \\&\mathcal Q^{\pi ^{\prime }}_1=\left\{ \left\{ \left( X,\left( {\frac{1}{2}},1\right) \right) ,\left( Y,\left( {\frac{1}{2}},0\right) \right) ,\left( X,\left( {\frac{1}{2}},{\frac{1}{2}}\right) \right) ,\left( Y,\left( {\frac{1}{2}},{\frac{1}{2}}\right) \right) \right\} \right\} , \\&\mathcal Q^{\pi ^{\prime }}_2=\left\{ \left\{ \left( X,\left( {\frac{1}{2}},1\right) \right) \right\} , \left\{ \left( Y,\left( {\frac{1}{2}},0\right) \right) \right\} ,\left\{ \left( X,\left( {\frac{1}{2}},{\frac{1}{2}}\right) \right) ,\left( Y,\left( {\frac{1}{2}}, {\frac{1}{2}}\right) \right) \right\} \right\} . \end{aligned}$$

It holds that $\mathcal Q^{\pi }_1$ is a finer partition than $\mathcal Q^{\pi ^{\prime }}_1$ and that $\mathcal Q^{\pi }_2=\mathcal Q^{\pi ^{\prime }}_2$. Thus, $\pi$ is more informative than $\pi ^{\prime }$. $\triangle$

While we do not require $\mathcal Q^{\pi }_i$ and $\mathcal Q^{\pi '}_i$ to be partitions to compare $\pi$ and $\pi '$, if they are partitions, then $\pi '$ is more informative than $\pi$ if for all $i \in N$ the restriction of $\mathcal Q_i^{\pi }$ to $\Lambda ^{\pi '}$ is at least as coarse as $\mathcal Q_i^{\pi '}$ and for some $i \in N$ the partitions $\mathcal Q^{\pi }_i$ and $\mathcal Q^{\pi '}_i$ are not the same.

### Proposition 9.5

Fix a set of message profiles *S*. Let $\sigma \in \Sigma (S)$, $\pi , \pi ' \in \Pi (\sigma )$, and $\pi \in \Pi ^\mathrm{{i}}(S)$. Then $\pi '$ is at least as informative as $\pi$ if and only if $\Lambda ^{\pi '}\subseteq \Lambda ^{\pi }$.

### Proof

Suppose that $\Lambda ^{\pi '}\setminus \Lambda ^\pi \ne \emptyset$. Then there exist $i\in N$ and $Q'\in \mathcal Q^{\pi '}_i$ such that there is no $Q\in \mathcal Q^\pi _i$ with $Q'\subseteq Q$. Hence, condition (*ii*) of Definition 9.2 is not satisfied and $\pi '$ is not at least as informative as $\pi$.

Now let $\Lambda ^{\pi '}\subseteq \Lambda ^{\pi }$. By Corollary 8.10 and Lemma 9.1 we can assume without loss of generality that $\pi \in \Pi ^\ell (S)$, so that $\mathcal Q_i^{\pi }=\mathcal P_i^{\pi }$ for all $i\in N$.

We first show Condition (ii) of Definition 9.2. So, let $i \in N$ and assume $Q\in \mathcal Q_i^{\pi }$ and $Q'\in \mathcal Q_i^{\pi '}$ are such that $Q\cap Q'\ne \emptyset$. We have to show that $Q'\subseteq Q$. Let $\left( \omega ^*,\lambda ^*\right) \in Q\cap Q'$. There is $\left( \omega ,\lambda \right) \in H^{\pi }$ such that $Q=Q_i^{\pi }\left( \omega ,\lambda \right) =P_i^{\pi }\left( \omega ,\lambda \right)$. Thus, by Lemma 8.3, we have that $Q = P_i^{\pi }\left( \omega ^*,\lambda ^*\right) .$ Consider $\left( \bar{\omega },\bar{\lambda }\right) \in Q'.$ There is $\left( \omega ',s'\right) \in H^{\pi '}$ such that $Q'=Q_i^{\pi '}\left( \omega ',s'\right)$ and there is $\left( \omega '',s''\right) \in P_i^{\pi '}\left( \omega ',s'\right)$ with $\lambda ^{\pi ',s''}=\bar{\lambda }$. In particular, since $s''_i=s'_i$, we have $\bar{\lambda }_i=\lambda _i^{\pi ',s''}=\lambda _i^{\pi ',s'}=\lambda _i^*$. Since $\Lambda ^{\pi '}\subseteq \Lambda ^{\pi }$, we have $\left( \bar{\omega },\bar{\lambda }\right) \in \Lambda ^{\pi }$, and since $\bar{\lambda }_i=\lambda _i^*$, we have $\left( \bar{\omega },\bar{\lambda }\right) \in P_i^{\pi }\left( \omega ^*,\lambda ^*\right) =Q$. We have shown that $Q' \subseteq Q.$

To prove Condition (i) of Definition 9.2 it is now sufficient to show that for each $Q'\in \mathcal Q_i^{\pi '}$ there is $Q\in \mathcal Q_i^{\pi }$ with $Q\cap Q'\ne \emptyset$. Let $\left( \omega ',s'\right) \in H^{\pi '}$ be such that $Q' = Q_i^{\pi ^{\prime }}\left( \omega ',s'\right) .$ It holds that $(\omega ', \lambda ^{\prime ^{s'}}) \in Q'\subseteq \Lambda ^{\pi '}\subseteq \Lambda ^{\pi }$. Thus, there is $Q \in \mathcal Q_i^{\pi }$ with $(\omega ', \lambda ^{\prime ^{s'}}) \in Q.$ $\square$

Proposition 9.5 reveals that among those signals that induce the same distribution over posterior belief profiles, those that are individually minimal and have the largest number of posterior histories are the least informative. We can interpret the condition $\Lambda ^{\pi '}\subseteq \Lambda ^{\pi }$ as $\pi '$ providing additional information about what posterior histories are impossible. It is worth mentioning that this condition together with the individual minimality of $\pi$ implies that $\mathcal Q_i^{\pi '}$ contains at least the same number of elements as $\mathcal Q_i^{\pi }$ and that these elements are smaller in the sense of set inclusion.

In Corollary 6.10 a signal is transformed into an LIS that induces the same distribution over posterior vectors. Although they are not equivalent if $\pi$ is not individually minimal, they have the same set of posterior histories as the next lemma shows.

### Lemma 9.6

Fix a set of message profiles *S*. Let $\Delta (\Omega )^{n} \subseteq S$ and $\pi \in \Pi (S).$ For $\pi ^{\ell }$ as defined in (7) it holds that $\Lambda ^{\pi ^\ell }=\Lambda ^{\pi }$.

### Proof

Observe that $\left( \omega ,\lambda \right) \in \Lambda ^{\pi }$ if and only if there is $s\in S^{\pi }$ such that $\lambda =\lambda ^{\pi ,s}$ and $\pi \left( s|\omega \right) >0$. This, however, is equivalent to $\pi ^{\ell }\left( \lambda |\omega \right) =\sum _{s\in S^{\pi }:\lambda ^{\pi ,s}=\lambda } \pi \left( s|\omega \right) >0,$ which holds if and only if $\left( \omega ,\lambda \right) \in H^{\pi ^{\ell }}=\Lambda ^{\pi ^{\ell }}$. $\square$

Proposition 9.5 and Lemma 9.6 immediately imply the following result.

### Corollary 9.7

Fix a set of message profiles *S*. Let $\Delta (\Omega )^{n} \subseteq S,$ $\pi \in \Pi (S),$ and $\pi ^{\ell } \in \Pi ^\ell (S)$ as defined in (7). Then $\pi$ is at least as informative as $\pi ^{\ell }$.

Corollary 9.7 might suggest that language-independent signals reveal as little information as possible. The following example demonstrates that this is, in general, not true.

### Example 9.8

Recall $\pi$ and $\pi '$ from Example 7.6. Both signals are language-independent and, hence, individually minimal. However, as shown in Example 8.1, $\Lambda ^{\pi '} = H^{\pi '}\subsetneq H^{\pi } = \Lambda ^{\pi }.$ Thus, by Proposition 9.5, $\pi '$ is more informative than $\pi$. Observe that it is not relevant that $\pi$ is an LIS: when translating each message sent under $\pi$ in two different languages and sending both with equal probability, we obtain a signal that is not even minimal, but equally informative as $\pi .$ $\triangle$

Our final result identifies those signals that are least informative. Let $\sigma \in \Sigma (S)$ and recall that the set $P(\sigma )$ is convex. The *relative interior* of $P(\sigma )$ is defined as

$$\begin{aligned} \text {relint}P(\sigma )=\left\{ p\in P(\sigma ) \vert \,\forall p'\in P(\sigma ),~\exists \alpha >1,~\alpha p+(1-\alpha )p'\in P(\sigma ) \right\} . \end{aligned}$$

### Proposition 9.9

Fix a set of message profiles *S*. Let $\Delta (\Omega )^{n} \subseteq S$ and $\sigma \in \Sigma (S)$. For every $p \in P(\sigma ),$ define the signal $\pi ^{p} \in \Pi ^{\ell }(S)$ by

$$\begin{aligned} \pi ^{p}\left( \lambda \vert \omega \right) =\frac{p(\omega ,\lambda )}{\lambda ^0(\omega )}, \quad \forall \omega \in \Omega ,~\lambda \in \rm{supp}(\sigma ). \end{aligned}$$

If $p\in \text {relint}P(\sigma )$, then every $\pi \in \Pi (\sigma )$ is at least as informative as $\pi ^p.$

### Proof

First observe that for every $p\in \text {relint}P(\sigma )$ it holds that $p\left( \omega ,\lambda \right) >0$ whenever there is $p'\in P\left( \sigma \right)$ with $p'\left( \omega ,\lambda \right) >0$. Thus, for any such $p,p'$ it holds that

$$\begin{aligned} \Lambda ^{\pi ^{p'}} = \{(\omega ,\lambda ) \in \Omega \times \text {supp}(\sigma ) | p'(\omega ,\lambda )> 0\} \subseteq \{(\omega ,\lambda ) \in \Omega \times \text {supp}(\sigma ) | p(\omega ,\lambda ) > 0\} = \Lambda ^{\pi ^p}. \end{aligned}$$

So, by Proposition 9.5, it holds that $\pi ^{p'}$ is at least as informative as $\pi ^p.$

Let $\pi \in \Pi (\sigma )$ and define $\pi ^{\ell } \in \Pi ^{\ell }(S)$ as in (7). Define $p^{\prime } \in P(\sigma )$ by

$$\begin{aligned} p^{\prime }(\omega ,\lambda ) = \lambda ^{0}(\omega ) \pi ^{\ell }(\lambda | \omega ), \quad \forall \omega \in \Omega ,~\forall \lambda \in \text {supp}(\sigma ). \end{aligned}$$

Then $\pi ^{\ell }=\pi ^{p'}$. Thus, as seen before, $\pi ^{\ell }$ is at least as informative as $\pi ^p$. Moreover, by Corollary 9.7, $\pi$ is at least as informative as $\pi ^{\ell }$. Hence, $\pi$ is at least as informative as $\pi ^p$. $\square$

Given a distribution $\sigma \in \Sigma (S),$ if *p* is in the relative interior of $P(\sigma ),$ then $\pi ^p$ is a least informative signal that induces $\sigma$. As $\Pi ^\ell (\sigma )$ is but a positive linear transformation of $P\left( \sigma \right)$, Proposition 9.9 implies that any signal $\pi \in \rm{relint}\Pi ^\ell (\sigma )$ is a least informative signal.

Recall signals $\pi$ and $\pi '$ from Example 7.6. We concluded in Example 9.8 that $\pi '$ is more informative than $\pi$. The result also follows from Proposition 9.9 as $p\in \text {relint}P(\sigma )$ and, hence, $\pi$ is a least informative signal.

## Conclusion

We consider a group of receivers who share a common prior on a finite state space and investigate (*i*) the inducible distributions of posterior belief profiles and (*ii*) the informativeness of signals. The sender can restrict attention to particular classes of signals without loss of generality. In particular, any distribution over posterior belief profiles can be induced by a language-independent signal. Moreover, any individually minimal signal can be transformed into an equivalent LIS.

Extending Kamenica and Gentzkow (2011) by allowing for multiple receivers and private communication imposes further constraints on inducible distributions over posterior belief profiles, so that Bayes plausibility is no longer a sufficient condition. We formulate the additional conditions in the form of a linear system of equations that needs to have a non-negative solution. These conditions, together with Bayes plausibility, are necessary and sufficient.

We define informativeness in terms of knowledge about the true *posterior history*. For every signal there is a language-independent signal that is not more informative. Any element in the relative interior of the set of all language-independent signals which induce a particular distribution belongs to the set of least informative signals.

One potential extension of the model would be to allow for distributions with countably infinite support. While some of our results would survive this extension, it is not immediately clear, for example, how one would define minimal or individually minimal signals, since their current definitions rely on distributions having finite support. Therefore, this provides an interesting topic for future research.

## Footnotes

1 This is also known as the martingale property.

2 This is a well-known example, e.g. Arieli et al. (2021) consider a sender who wishes to maximize the distance between two receivers’ beliefs.

3 All these papers were developed independently from ours and vice versa and written roughly around the same time.

4 Li (2017) considers a different criterion and measures informativeness in the sense of Ganuza and Penalva (2010), where more informative message profiles lead to greater variability of conditional expectations.

5 Countably infinite support might be important in some information design applications. We would like to suggest the extension of our results to distributions with countably infinite support as an interesting direction for future research.

6 In this paper, we are explicit about the set of messages that are available to sender. Thus, in our results we will provide appropriate conditions on the sets $S_i$ whenever needed.

7 Recall that $\Delta (X)$ is defined as the set of distributions over *X* with finite support and note that if $\lambda$ is such that there is no *s* with $\lambda =\lambda ^{\pi ,s}$, then the right-hand side of (2) is 0.

8 Koessler et al. (2022) adopt a similar definition for inducible distributions called *splittings* and employ it to characterize Bayes–Nash equilibria of an information design game with multiple senders.

9 Observe that this is no contradiction to the proof of Proposition 3.1: there we used that any fixed message induces under every signal where it is sent with positive probability the same posterior. Here, message *x* induces posterior $\left( 1/2,1/2\right)$ under $\pi$ but $\left( 1/4,3/4\right)$ under $\pi '$.

10 Observe that Proposition 6.5 can be reformulated to allow for a finite set *S*. In particular, given a finite *S*, for $\pi \in \Pi (S)$ with $\sigma ^\pi \in \Sigma (S)$ and $\text {supp}\left( \sigma ^\pi \right) \subseteq S$ it holds that $\Pi ^\ell \left( \sigma ^\pi \right)$ is non-empty and convex.

11 The relation $\sim$ is reflexive, symmetric, and transitive, so is an equivalence relation.

12 It is implied by the proof of Lemma A.2  in Kerman et al. (2023).

13 Other papers have also pointed this out and provided examples, see, e.g. Arieli et al. (2021).

14 Note that $$0\in \mathbb R_+$$.

15 Morris (2020) provides an alternative proof for the no trade result that also applies to a finite state space.

16 Note that an alternative definition of $Q^\pi _i$ is to employ $\Lambda ^\pi$ also as the domain. However, since the (first-order) posterior does not contain any information about higher order beliefs (which is encoded in messages), two posterior correspondences that are different according our definition of $Q^\pi _i$ could be the same under the alternative one, e.g. $Q^\pi _1$ and $Q^{\pi '}_1$ in Example 8.6 would be equal.
